# The Role of the Extracellular Matrix and Its Molecular and Cellular Regulators in Cancer Cell Plasticity

**DOI:** 10.3389/fonc.2018.00431

**Published:** 2018-10-09

**Authors:** Valentina Poltavets, Marina Kochetkova, Stuart M. Pitson, Michael S. Samuel

**Affiliations:** ^1^Centre for Cancer Biology, SA Pathology and University of South Australia, Adelaide, SA, Australia; ^2^Adelaide Medical School, Faculty of Health Sciences, University of Adelaide, Adelaide, SA, Australia

**Keywords:** extracellular matrix, stroma, plasticity, cancer associated fibroblasts (CAF), tumor associated macrophages, tumor microenvironment, signaling pathways, cancer

## Abstract

The microenvironment encompasses all components of a tumor other than the cancer cells themselves. It is highly heterogenous, comprising a cellular component that includes immune cells, fibroblasts, adipocytes, and endothelial cells, and a non-cellular component, which is a meshwork of polymeric proteins and accessory molecules, termed the extracellular matrix (ECM). The ECM provides both a biochemical and biomechanical context within which cancer cells exist. Cancer progression is dependent on the ability of cancer cells to traverse the ECM barrier, access the circulation and establish distal metastases. Communication between cancer cells and the microenvironment is therefore an important aspect of tumor progression. Significant progress has been made in identifying the molecular mechanisms that enable cancer cells to subvert the immune component of the microenvironment to facilitate tumor growth and spread. While much less is known about how the tumor cells adapt to changes in the ECM nor indeed how they influence ECM structure and composition, the importance of the ECM to cancer progression is now well established. Plasticity refers to the ability of cancer cells to modify their physiological characteristics, permitting them to survive hostile microenvironments and resist therapy. Examples include the acquisition of stemness characteristics and the epithelial-mesenchymal and mesenchymal-epithelial transitions. There is emerging evidence that the biochemical and biomechanical properties of the ECM influence cancer cell plasticity and vice versa. Outstanding challenges for the field remain the identification of the cellular mechanisms by which cancer cells establish tumor-promoting ECM characteristics and delineating the key molecular mechanisms underlying ECM-induced cancer cell plasticity. Here we summarize the current state of understanding about the relationships between cancer cells and the main stromal cell types of the microenvironment that determine ECM characteristics, and the key molecular pathways that govern this three-way interaction to regulate cancer cell plasticity. We postulate that a comprehensive understanding of this dynamic system will be required to fully exploit opportunities for targeting the ECM regulators of cancer cell plasticity.

## Introduction

**Figure 1:**
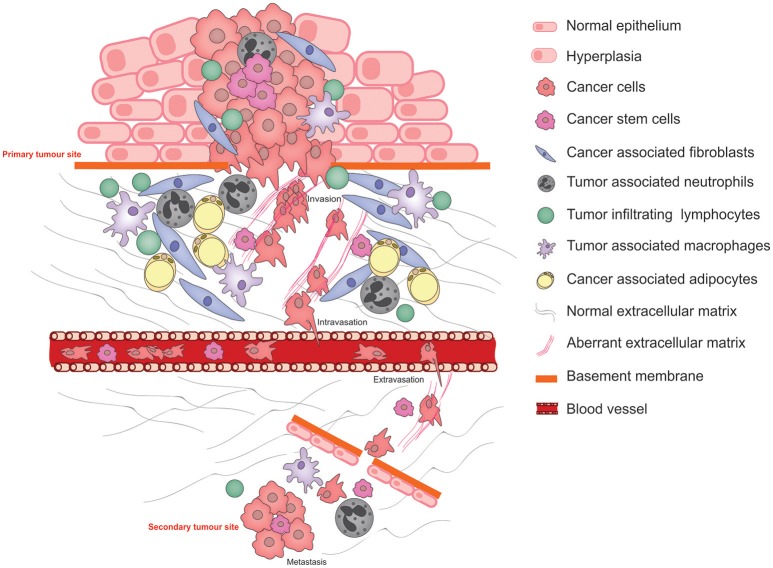
Schematic illustrating the pathophysiological processes that exploit cancer cell plasticity during tumor progression, invasion, and metastasis.

Metastasis is the primary cause of cancer-related mortality (1) and results in a catastrophic disruption to an organ function through the lodgment and unrestrained growth of exogenous tumor cells within normal tissue. For a tumor cell to migrate to a new location within the body, it needs to adapt to survive and thrive within an environment that is distinct from that of the tissue in which it arose. Functional adaptations acquired by cancer cells to survive altered environments is termed cancer cell phenotypic plasticity. Of these, the epithelial to mesenchymal transition (EMT) is the best studied and its reverse, the mesenchymal to epithelial transition (MET) is rather less well understood. Another key aspect of phenotypic cancer cell plasticity is the acquisition of stem-like characteristics, resulting from so-called de-differentiation, which permits the cancer cells to remain dormant for long periods of time, evading both the immune system and therapeutic agents. The pathophysiological processes of metastasis that require phenotypic cancer cell plasticity and the major cellular players that bring this about are summarized in Figure 1.

Normal interactions between the parenchyma and the stroma are characterized by (1) A two-way communication by molecular messengers that are secreted into the microenvironment, (2) biochemical and biophysical cues exerted by the ECM, and (3) direct cell-cell contact permitting reciprocal signaling between the two cell types. These interactions direct tissue homeostasis and the establishment of niches bearing distinct microenvironmental characteristics that facilitate the maintenance of specialized cell types including stem cells. Under abnormal conditions in which the parenchymal cells acquire tumor-causing genetic lesions, the microenvironment—its cellular and ECM components—is remodeled under the influence of the growing tumor as well as the organism, resulting in aberrant tissue homeostasis and disruption of the specialized niches. These microenvironment changes strongly influence the progression of the disease (2).

In cancer progression, epithelial-mesenchymal transition is associated with invasiveness and metastasis. Acquisition of a mesenchymal phenotype is characterized by increased motility, expression of ECM remodeling enzymes such as matrix metalloproteases (MMPs), and enhanced survival—all key adaptations that are required for traversing the basement membrane, promoting abnormal interactions between cancer cells and the extracellular matrix (ECM), intravasation and survival within the circulation. Conversely, MET is associated with integration into epithelia at sites of distal metastasis. EMT has long been associated with acquisition and maintenance of cancer stem cells (CSCs) (3).

The CSC hypothesis takes its origins from the observations made in the hematopoietic system, where a pluripotent progenitor gives rise to all hierarchical lineages of the system by a stepwise process of differentiation (4). Analogously, CSCs are thought to constitute a reserve pool of a limited number of cells that maintain the proliferative potential of the primary cancer or migrate out of the primary site to seed new secondary tumors at the metastatic sites. Recent observations have permitted a more nuanced understanding of CSCs. It has been reported that like bulk cancer cells, CSCs exhibit phenotypic plasticity in response to signals from the microenvironment environment (5). Another important addition to the emerging CSC model is that the microenvironment plays a crucial role for the maintenance of the CSC pool, just as it does for the maintenance of normal stem cells (6). However, context-specific differences between tumor types exist; for instance, while CSCs of colorectal cancer may be generated from non-CSC cells via a process regulated by Wnt signaling, a strictly hierarchical system is characteristic of glioblastomas, where, CSCs are maintained by self-renewal (7). There is strong circumstantial evidence that ECM provides an important stem cell niche given the dependence of normal stem cells on signaling through ECM receptors such as the laminin receptor, α6β1 integrin (8), the vitronectin receptor αV (9), and collagen receptors (10) and emerging evidence that the cancer-associated ECM is an important aspect of the cancer stem cell niche (11).

Cancer plasticity is driven by reciprocal interactions between a cancer cell and its microenvironment, which permits this cell to, on the one hand, calibrate its response to the altered environment and on the other, actively remodel the microenvironment to facilitate its survival and proliferation. In this review, we will discuss how the ECM influences cancer cell plasticity and conversely how cancer cells directly or indirectly influence changes in ECM structure and composition.

The extracellular matrix is a scaffold of fibrillar proteins, accessory proteins and molecules that provides structural and biochemical support for cells. The predominant component of the ECM is fibrillar collagen, the structure and mechanical properties of which strongly influence cellular phenotype (12). Based on biochemical and structural characteristics, the ECM consists of a basement membrane (located at the basal aspect of epithelial or endothelial cells in normal tissues) and the interstitial (stromal) ECM. In most tissues, the basement membrane consists largely of collagen IV, together with laminin, fibronectin, and several types of proteoglycans. The main role of the basement membrane is to provide a physical barrier between the epithelial cells and the connective tissue (stroma) of the organ, whilst still allowing the diffusion of gases and transport of signaling molecules. The interstitial ECM, mainly produced by mesenchymal cells (discussed further in section Cellular Mediators of Cancer Cell Plasticity via the ECM), consists largely of collagens I and III, fibronectin, and proteoglycans. In cancer, rupture of the basement membrane permits epithelial cells to undergo an EMT and migrate into the surrounding stroma and invade through the interstitial ECM. Epithelial cells that have undergone EMT can cause activation of stromal cells to yield pro-tumorigenic stromal cells that can remodel the ECM to create a tumor-permissive environment (13). Among the components of the ECM, glycosaminoglycans such as hyaluronan (HA) play important roles during cancer progression. High levels of HA have been documented in tumors and are associated with poor prognosis and chemotherapy resistance (14). HA has been shown to be able to induce EMT by binding to CD44 and activating the EMT transcription factor TWIST-1 (15). Increased HA levels have also been shown to compromise vascular integrity in tumors which has important implications for metastasis (16). Furthermore, HA breakdown products have been implicated in inflammatory responses that precipitate extracellular matrix remodeling (17).

The normal ECM is highly remodeled after it has been initially set down and exhibits tissue-specific composition and organization. In pathological conditions such as desmoplasia, the appearance of linear ECM fibers correlates with poor patient outcomes. Linear fibers have been observed to provide tracks that migratory cancer cells can use to their advantage (18), to enhance migratory capability. The main regulators of ECM remodeling during tumorigenesis are cancer-associated fibroblasts (CAFs), which produce large quantities of collagen I, fibronectin, and periostin (13). Analysis of the ECM using techniques such as second harmonic generation (SHG) microscopy, atomic force microscopy and mass spectrometry has revealed tissue-specific composition and configuration of its components, which underlie tissue phenotype, and also the tumor phenotype (19). The ECM is a source of biochemical and biomechanical signals that promote tumor progression, and it is in turn strongly influenced by the cancer in a reciprocal relationship that is driven by the cytoskeleton of cancer cells (20).

Cell-ECM interactions in both normal and pathologic conditions are principally mediated via integrins, which constitute a large family of cell-surface receptors. Integrins also regulate cytoskeleton organization and activate intracellular signaling pathways, conveying both mechanical and chemical signaling (21). Besides their roles in cell adhesion and migration, they also transmit signals for cell proliferation and survival. The majority of integrins activate focal adhesion kinase (FAK). This in turn promotes directional cell motility of both tumor and stromal cells, and generates signals to further modify ECM organization, thereby altering the mechanical properties of the tumor microenvironment (13, 21).

## Cellular mediators of cancer cell plasticity via the ECM

Normal tissue homeostasis is strongly influenced by the ECM and a key example of this is the process of wound healing. One of the steps for the re-establishment of normal tissue homeostasis following wounding is the migration of fibroblasts into the wound space in order to break down the thrombus and regenerate the ECM (22). The mechanical properties of the newly synthesized ECM are an important determinant of how quickly the wound heals (23). Similarly, the ECM is set down early in embryonic development and influences the delamination, migration and differentiation at their destination of diverse cell types (24, 25). Since the physiological functions and behaviors of normal cell types and strongly influenced by the normal ECM, it is no surprise that similarly the tumor ECM exerts a strong influence on the behavior or cancer cells. The influence of the ECM on cancer cell plasticity is modulated by a variety of cell types that reside within the tumor stroma. Under the influence of systemic regulators as well as cancer cells, these stromal cells not only produce tumor ECM, which qualitatively and quantitatively differs from a normal ECM, but also an array of cytokines and other secreted and membrane-bound factors that influence cancer cell plasticity. In this section, we discuss the key cellular mediators of cancer cell plasticity that regulate the biochemical and biomechanical properties of the ECM.

### Cancer-associated fibroblasts (CAFs)

Fibroblasts, a cell type of spindle-like morphology and mesenchymal lineage, constitute the major cell type of the normal tissue stroma. Stroma-resident fibroblasts that are not actively engaged in ECM production or turnover are termed “resting” or “quiescent.” Resting fibroblasts are mostly observed within fibrillar ECM and have the potential to be “activated.” Activated fibroblasts are morphologically and metabolically different to their resting counterparts, and activation can be caused by acute or chronic inflammatory responses such as wound healing or fibrosis. Pro-inflammatory factors such as TGF-β, IL-6, platelet-derived growth factor (PDGF), hypoxia, and reactive oxygen species (ROS) can activate quiescent fibroblasts. Once activated, fibroblasts synthesize and deposit ECM components, release chemokines and cytokines into the stroma and generate tissue-level tensile forces via their actomyosin cytoskeletons, all key requirements for tissue remodeling. Activated fibroblasts are therefore essential for epithelial cell differentiation, control of immune responses and the maintenance of tissue homeostasis (26, 27).

A long-standing concept tumor as “wounds that do not heal” (28) hinges on the ability of cancers to commandeer fibroblast function normally associated with wound healing to promote disease progression. Accumulation of tumor cells within the tissue can trigger chronic wound healing responses from normal tissue fibroblasts, leading to desmoplastic tissue remodeling characterized by the appearance of aberrantly organized ECM fibers and increased tissue stiffness, which in turn creates a favorable environment for tumor progression (29).

Activated fibroblasts in the tumor microenvironment are termed cancer-associated fibroblasts (CAFs). CAFs are among the main cellular contributors to cancer-associated changes in ECM architecture and may arise from normal fibroblasts. CAFs are thought to be recruited via growth factors secreted by tumor and immune cells (such as TGFβ, PDGF, and FGF2), and subsequent proliferation and expansion of these cells may be auto-regulated by paracrine/autocrine mechanisms governed by other CAF populations (27). There is an ongoing discussion regarding the classification of CAF populations based on cell morphology, markers, secretory profiles, and location within the tumor. These complex issues and the debate around the pro- vs. anti-tumorigenic properties of CAFs are dealt with in detail elsewhere (26, 27, 30). Here, we discuss mechanistic aspects of the contribution of CAFs and other stromal cells to the ECM properties that regulate cancer cell plasticity.

CAFs are among the few stromal cell types that have been conclusively shown to promote an EMT program in cancer cells. Using stromal fibroblasts isolated from breast cancer patients in co-culturing experiments with a panel of breast cancer cell lines, CAFs were demonstrated to promote cancer cell EMT via TGF-β secretion and induction of the TGF-β/SMAD signaling pathway in the cancer cells (31). Another study found that activated fibroblasts secrete carbonic anhydrase IX (CA IX), which enhances the production by CAFs of MMP2 and MMP9, which are well-known to degrade and remodel the ECM. Acidification of the microenvironment by CA IX can also directly promote an EMT program in prostate carcinoma cells (32). Furthermore, IL-6 from prostate carcinoma cells generates a CAF phenotype and leads to increased MMP2 and MMP9 levels in fibroblasts. This can in turn induce an EMT program in cancer cells. This reciprocal cancer cell-CAF interaction sustains tumor progression via cancer cell plasticity (33).

Recent evidence suggests that ECM remodeling components secreted by CAFs play a role in the maintenance of the cancer stem cell niche (34, 35). For example, mammary cancer cells can induce ECM periostin production by stromal fibroblasts, essential for CSC maintenance by promoting Wnt signaling (36). More recently, it has become clear that CAF phenotype changes induced by tumor-initiated hedgehog signaling promotes stemness in breast cancers in both mouse models and human patients and that inhibiting hedgehog signaling in fibroblasts may be a useful therapeutic modality to reverse breast cancer cell plasticity (37). These CAF functions are dependent on their role in regulating the ECM and these ECM changes occur at the site of the stem cell niche (37). Fibroblasts lacking Tissue Inhibitor of Metalloproteinases (TIMPs) exhibit a CAF-like phenotype and release extracellular vesicles packed with factors that enhance cancer cell motility and upregulate CSC markers. These vesicles contained high levels of A Disintegrin and Metalloproteinase domain containing protein 10 (ADAM10), which promotes cell motility via activation of RhoA and Notch signaling (38). CAF-derived growth factors were also shown to play a role in stem cell niche formation. CAF-derived HGF is proposed to promote the formation of the CSC niche and tumorigenicity by activating the Wnt signaling pathway in differentiated colon cancer cells (39). Another report suggests that CAFs promote growth and stemness in lung CSCs. Paracrine signaling between CAF-derived insulin-like growth factor-II (IGF-II) and IGF1R on CSCs, and the subsequent induction of Nanog, induced expression of CSC markers. The importance of this signaling axis was also confirmed in samples from non-small cell lung cancer (NSCLC) patients (40). Taken together, these observations establish a key role for CAF-mediated ECM production and remodeling in cancer cell plasticity that promotes tumor progression.

It is now well-accepted that cancer cell motility is enhanced by the tumor ECM (41). It has been shown that TGF-β-stimulated colon CAFs are able to secrete scatter factor/hepatocyte growth factor (SF/HGF) and tenascin C, and thereby promote invasiveness of colon cancer cells (42). Using fibroblasts isolated from different stages of mouse mammary tumors it has been shown that activation of Yes-associated protein 1 (YAP1) in CAFs promotes matrix stiffening, cancer cell invasion, and angiogenesis. YAP1 is known to regulate cytoskeletal components including the regulatory myosin light chain (MLC2), which controls actomyosin contractility. A feed-forward loop is therefore established via the activation of YAP1 in response to mechanical cues from the ECM upon CAFs, which further stiffen of the ECM (43). Consistent with these observations, ROCK inhibition upstream of YAP1 reversed the CAF phenotype to normal (43). However, there are multiple pathways contributing to this feed-forward loop as ROCK-dependent actomyosin contractility downstream of GP130-IL6 JAK1/STAT pathway activation also enhanced ECM remodeling by CAFs, which in turn promoted melanoma cell migration *in vitro* (44). Therefore, the ability of fibroblasts to promote tumor cell migration while also enhancing tumor cell plasticity establishes a key role for this versatile cell-type in tumor progression.

Fibroblasts therefore exhibit key properties that are exploitable by cancer cells to promote tumor progression via cellular plasticity and interfering with CAF function therefore represents an attractive possibility for anti-cancer therapy. Nevertheless, evidence that at least a sub-population of CAFs has anti-tumor functions sounds a note of caution, raising the possibility that directly targeting CAFs may have unintended consequences. These observations highlight that more work needs to be done to dissect out the mechanisms by which CAFs contribute to cancer, with tissue- and context-dependent implications being likely to arise.

### Tumor-associated macrophages (TAMs)

Macrophages are phagocytic cells of the immune system that are distributed throughout virtually all tissues. They are highly adaptable cells that exhibit a high degree of plasticity depending on the signals in their immediate environment (45). In response to infection or injury, macrophages can secrete pro-inflammatory factors (TNF-α, IL-1, and nitric oxide) that trigger host defense responses and tissue remodeling. In tissue repair responses, an important switch occurs between pro-inflammatory and anti-inflammatory macrophage sub-populations. If not checked, the pro-inflammatory responses can lead to chronic inflammation or auto-immune disease (46). Not only are macrophages important contributors to innate immunity, but they also play essential roles in various developmental processes such as bone morphogenesis, neuronal patterning, angiogenesis, branching morphogenesis, and adipogenesis (47). These functions are co-opted by tumor cells as a feature of many cancers.

An important concept in macrophage biology is polarization; the phenotyping of macrophages based upon the expression of distinct suites of surface markers induced by specific environmental stimuli (48). While there has been a consensus on a two category “M1-M2” classification, it is now commonly accepted that macrophages exist on a continuum in disease and tissue specific contexts, of which the M1 and M2 states represent two extremes (45, 49). Macrophages polarized toward the M1 state are referred to as “classically” activated. This population produces pro-inflammatory agents that contribute to host defense and their anti-tumor properties. Macrophages polarized toward the M2 state are said to be “alternatively” activated. They secrete anti-inflammatory cytokines that largely suppress inflammatory responses. This population suppresses tumor immunity, enhances tumor angiogenesis, and extracellular matrix remodeling, and is associated with wound healing (47). Tumor-associated macrophages are also sometimes referred to as M2 polarized, although even in this context, heterogeneous populations of TAMs can exist within the M1-M2 continuum (50).

The specific location of TAMs within a tumor has been established as an important indicator of their pro-tumor activity, and they are mainly localized to perivascular regions or at the tumor invasive front. Monocytes are recruited to the invasive front and differentiate into macrophages in response to signals from tumor and stromal cells. An array of cytokines (IL-4, IL-10, IL-13), chemokines (CCL2, CXCL12), and growth factors (CSF-1, TGF-β, VEFG-A, PDGF, angiopoietin-2) produced at the invasive margin stimulate monocyte recruitment, differentiation and survival (51–54). We have previously demonstrated that the chemokine receptor CCR6 is expressed on TAMs and facilitates their migration to the cancer site in a mouse model of mammary cancer. Deletion of this chemokine receptor significantly decreases the population of TAMs, in particular M2 TAMs, as well as tumor burden (55).

TAMs play important roles in cancer cell proliferation (56), invasion (57), angiogenesis (58), and metastasis (45). TAMs secrete EGF, FGFs, and VEGFs that promote tumor cell proliferation, fibroblast activation and angiogenesis (59, 60). TAMs also produce IL-10 and TGF-β, which contribute to their immune-suppressive properties, assisting tumor cells in immune evasion (51, 61, 62). Chemotaxis-based experiments and intravital imaging revealed that reciprocal signaling between tumor cell-derived CSF-1 and TAM-derived EGF is essential for the promotion of tumor cell migration. This interaction is important for EGF receptor-mediated mammary tumor cell invasion in primary tumors (51). Furthermore, direct physical interaction between mammary cancer cells and TAMs has been observed using multiphoton intravital imaging, demonstrating that these reciprocal interactions may not only be biochemical in nature. The observation that tumor cells intravasate into areas where perivascular macrophages are numerous in mammary tumors, suggests that macrophages may also enhance cancer cell intravasation (63).

Along with their important roles in initiating growth and immune-suppressive signals directly, TAMs have been shown to play a significant role in contributing to the tumor ECM by producing several important matrix and matrix-associated proteins such as collagens, fibronectin, osteopontin, and periostin (64). Utilizing an orthotopic colorectal cancer (CRC) model, Afik and colleagues demonstrated that TAMs are capable of collagen synthesis and deposition, particularly collagen types I, VI, and XIV. Confocal, second harmonic generation and scanning electron microscopy of *ex vivo* mouse colorectal tumor tissues has revealed that TAMs are capable of initiating deposition, cross-linking, and linearization of collagen fibers during tumor development, particularly at the invasive front (65).

TAMs support tumor cell migration, invasion and metastasis via ECM remodeling (64, 66). Responding to cytokine signals from tumor cells, TAMs are known to secrete a cocktail of ECM remodeling enzymes including MMPs (1, 9, 12, and 14), serine proteases, cathepsins (B, S, C, L, Z), lysosomal enzymes, and ADAMs. These proteolytic enzymes disrupt integrin-mediated cell-cell adhesions and are essential for cancer cell invasion. In another study, TAMs isolated from breast cancers were observed to secrete CCL18, which signals via the breast cancer cell-specific PITPNM3 receptor. This signaling cascade activates integrin clustering on tumor cells, promoting integrin-ECM interactions and adhesion, thereby promoting invasiveness and metastasis (67). This study provides evidence for an orchestrated sequence of events whereby proteases released by TAMs remodel the ECM to facilitate tumor cell interaction while also releasing CCL18 that causes integrin clustering on tumor cells, strengthening cell-ECM interactions and facilitating cancer cell plasticity, migration, and dissemination.

Another important role for matrix remodeling enzymes secreted by TAMs is their ability to liberate the ECM-bound growth factors and signaling molecules that can influence tumor cell growth, plasticity, and motility (64). Liberation of bioactive fragments of ECM proteins (such as endostatin from type XVIII collagen) (68) that exhibit biological activities that are distinct from their parent ECM molecule was also demonstrated to be brought about by TAMs. Whilst this is an emerging area of TAM biology, it is one that is likely to increase in interest and significance.

There is a substantial evidence for a role for TAMs in promoting EMT in tumor cells through multiple mechanisms. Exposure of either mouse F9-teratocarcinoma or mammary epithelial cells to TAM-conditioned medium reduces E-cadherin expression, activates the Wnt/β-catenin pathway, induces the expression of mesenchymal markers and increases invasiveness of epithelial cells. It is also suggested that TAM-produced TGF-β may induce an EMT program in cancer cells (62). TAMS have been shown to induce an EMT program in pancreatic cancer cells in response to TLR4 signaling by producing IL-10 (69), and in a breast cancer model, TAMs induced EMT in cancer cells via upregulation of CCL18 (70). Even though the evidence points to a role for TAMs in EMT, it is becoming increasingly apparent that TAM-mediated EMT induction is context dependent and that microenvironmental factors determine the mechanisms by which TAMs induce cancer EMT programs. Analogous to this process, there is some evidence that TAMs are involved in cancer stem cell maintenance. Multiple studies have shown that growth factors and cytokines secreted by TAMs can promote and maintain the CSC populations within various tumors (71). Interestingly, in hepatocellular carcinoma, TAM-derived TGF-β1 promoted cancer cell stemness (72). Taken together, these observations provide evidence for a role for TAMs in ECM-dependent and ECM-independent regulation of tumor cell plasticity.

### Tumor-associated neutrophils (TANs)

Neutrophils, the most abundant leukocyte type in the blood, are produced in the bone marrow from hematopoietic stem cells and are released into circulation as fully mature cells. The generation and maturation of neutrophils is a complex process (73, 74) and is primarily regulated by granulocyte-colony stimulating factor (G-CSF). Other factors, such as granulocyte–macrophage-colony stimulating factor (GM-CSF), interleukin 6 (IL-6), and KIT ligand (KITL) also contribute to the production of neutrophils. In cancer, tumor cells secrete G-CSF which causes neutrophil overproduction, contributing to immunosuppressive responses at the early stages of tumorigenesis (75).

In the process of neutrophil maturation, primary, secondary, and tertiary cytoplasmic granules are formed. These pre-formed granules contain a wide variety of proteins and enzymes that are essential for anti-microbial defense and the resolution of inflammation. MMPs and neutrophil elastase contained within these granules are of interest as they are proteolytic enzymes that promote tumor progression by remodeling the cancer ECM (76_–_78).

Like fibroblasts and macrophages, neutrophils also exhibit polarization. Anti-tumor neutrophil populations are designated “N1” and pro-tumor as “N2.” Polarization toward the N2 form is induced by elevated levels of TGF-β, and N2-polarized neutrophils express high levels of CXCR4, VEGF, and MMP9. Blocking TGF-β in the microenvironment stimulates upregulation of TNFα and IFNγ in N1 neutrophils and causes CXCL2, CXCL5, and CCL3 production that leads to further recruitment of neutrophils to the tumor site (79). It was also shown that keratinocyte-derived TNF-α is an important contributor to early recruitment of neutrophils in a mouse cutaneous carcinoma model (80). Factors secreted by tumor cells also mediate recruitment of neutrophils. Using orthotropic transplantation of human hepatocellular carcinoma (HCC) cell lines into nude mice, Zhou et al. identified that CXCL5 secreted by cancer cells promotes neutrophil recruitment. Importantly, correlation between the levels of CXCL5 and neutrophil infiltration was confirmed in three independent clinical HCC patient cohorts (81).

Tumor-promoting properties of neutrophils have been documented and several of these functions involve ECM remodeling and cancer cell plasticity. Neutrophil-derived MMP9 enables keratinocyte hyperproliferation and invasiveness in a virus-induced cutaneous carcinoma model (82). In orthotopic xenograft transplantation systems of human fibrosarcoma and prostate carcinoma cell lines, tumor-recruited neutrophils release MMP9 that remodels the ECM to induce angiogenesis and promote metastasis (83).

Neutrophils have also been implicated in cancer cell EMT. Neutrophil-derived elastase has been shown to cleave E-cadherin and induce an EMT program in pancreatic ductal adenocarcinoma (PDAC) cells in co-culture with macrophages. Accordingly, in human PDAC tissue samples, EMT correlated with the presence of infiltrating neutrophils (84). In a zebrafish model, oncogene-transformed keratinocytes were shown to recruit neutrophils to enhance their EMT program. This process was mediated by signaling through CXCR2 in neutrophils (85), consistent with the observation that neutrophil recruitment and tumor progression are impaired in Cxcr2-deficient mice in several models of carcinoma (86). In a zebrafish xenograft model of tumorigenesis *in vivo*, neutrophil migration enhanced tumor cell invasion due to the establishment of collagen tracks that were exploited by cancer cells for their migration (87). Several lines of evidence therefore suggest that neutrophils modify the ECM to promote tumor progression with at least a proportion of these functions mediated by tumor cell plasticity.

Emerging evidence suggests that neutrophil-mediated ECM remodeling augments tumor invasiveness. Co-culture experiments of oral squamous cell carcinoma (OSCC) cell lines with neutrophils revealed that neutrophils increase the formation of invadopodia and collagenous matrix degradation by cancer cells. This process was induced via IL-8-mediated recruitment of neutrophils and subsequent release of TNF-α by neutrophils into the surrounding microenvironment (88). Consistent with these observations, a transgenic mouse mammary cancer model exhibited distinct cytokine profiles in collagen-dense tumors compared to low collagen-density tumors and these cytokine profiles were associated with neutrophil maturation in collagen-dense cancer tissues. Accordingly, depletion of neutrophils in collagen-dense mammary tumors reduced tumor progression in collagen-dense tumors (89).

Another intriguing field that has recently emerged is the study of neutrophil extracellular traps (NETs) and their contribution to tumor progression. NETs are three-dimensional networks of extruded DNA packed with cytosolic and granule proteins. NETs were first described as contributors to the innate immune response, with an ability to trap extracellular pathogens. It has since been shown that inflammatory responses can trigger NET formation (or NETosis). Comprehensive reviews on the roles of NETs in tumorigenesis have been recently published (90, 91). For the purposes of this review we will focus our attention on the potential contribution of NETs to regulation of ECM composition in the tumor microenvironment. NET components MMP9, cathepsin G and neutrophil elastase are all known to contribute to extracellular matrix remodeling as well as provide signals for tumor cell proliferation, migration and tumor-associated angiogenesis (91). While it is yet to be determined whether these proteins contribute to ECM remodeling in the cancer microenvironment while associated with NETs, there is *in vitro* evidence that they may. One study has demonstrated the ability of NETs to trap cancer cells under static and dynamic conditions, raising speculation that NETs produced during inflammation could assist in the colonization of secondary tissues by circulating cancer cells (92). Another recent study has demonstrated that cell lines generated from chronic myelogenous leukemia use integrins to adhere to the fibronectin in NETs. It is therefore possible that NETs provide cancer cells with a platform for interaction with other cells and can induce key signaling pathways required for their survival and proliferation (93). Further investigation into the role of NETs in ECM remodeling, and contribution of NET formations to desmoplastic response in cancers, is therefore warranted. Taken together these studies suggest that new roles for neutrophils in ECM biology are likely to be uncovered, and thereby a role in regulating cancer cell plasticity.

### Cancer-associated adipocytes (CAAs)

Adipocytes are the lipid-storing cells of adipose tissues (AT) that regulate energy storage and metabolism within the body. Adipocytes secrete hormones and other molecules, collectively termed adipokines, which exert paracrine and endocrine regulatory roles in obesity, adipose tissue fibrosis, inflammation, tumorigenesis, and cancer metabolism (94–96). Many studies indicate a clear phenotypic difference between CAAs and normal adipocytes, but most studies investigating the roles of adipokines in cancer rely on mature (differentiated) adipocyte co-culture experiments with cancer cells. In the context of the tumor microenvironment, the role of adipokines is more complex than simple reciprocal interactions between adipocyte and tumor cells—even though tumor cells express corresponding receptors for adipokines—and is likely to also be strongly influenced by the inflammatory milieu.

Adipocytes mainly arise from mesenchymal stem cells (MSCs) or undifferentiated adipocyte precursors within adipose tissue stroma (97, 98). A small proportion of adipocytes can also be derived from hematopoietic stem cells (HSCs) (99, 100). Adipocytes constitute an essential cellular component of the tumor microenvironment in breast, ovarian, prostate, renal, gastric, and colon cancers (96). Tumor cells can “activate” adipocytes and subvert their cellular programs to facilitate tumor-promotion. Such activated cancer-associated adipocytes are distinct from normal adipocytes in morphology and function. Adipocytes co-cultured with cancer cells exhibit de-lipidation, decreased expression of adipocyte markers such as Ap2 and FABP4, increased expression of MMP11, and enhanced release of inflammation-promoting cytokines IL-6 and IL-1β. Importantly, presence of CAAs expressing IL-6 was confirmed *ex vivo* using primary breast cancer samples (101). Co-culture of cancer cells with mature adipocytes can induce adipocyte dedifferentiation via the Wnt/β-catenin pathway. Adipocytes shrink, significantly lose their lipid content, and may acquire fibroblast-like properties. These cells, termed adipocyte derived fibroblasts (ADFs), express the fibroblast marker S100A4/FSP-1 but not α-SMA. ADFs acquire migratory capacity and move toward the tumor core to promote cancer cell invasion (102).

There is evidence that mature adipocytes, CAAs and ADFs contribute to tumor cell plasticity. Mature human breast adipocytes increase *in vitro* cell motility of both pre-malignant and malignant breast cancer cell lines (103). Through lipolysis and direct lipid transfer from adipocytes to cancer cells, adipocytes may serve as energy reservoirs for cancer cells and sustain tumor growth (104). *In vitro* studies show that paracrine signaling from cancer cells induces the release of free fatty acids from CAAs resulting in CAA de-lipidation and increased secretion of inflammatory cytokines and proteases that promote tumor cell invasiveness (105).

An intriguing discussion is now underway regarding the role of obesity-mediated changes in the tumor microenvironment and cancer progression (106). Obesity has been implicated in the promotion of inflammation and fibrosis, particularly through the engagement of hypoxia-induced transcriptional programs in adipocytes and the subsequent recruitment of immune cells. In mouse models of spontaneous pancreatic ductal adenocarcinoma (PDAC) it was shown that adipocyte-mediated inflammation contributed to a desmoplastic response through the recruitment of TANs, which enhanced tumor formation in obese animals (107). It has also been demonstrated that mammary adipose tissue in obese mice contained larger myofibroblast populations than in lean counterparts and that these myofibroblast populations contributed to ECM stiffness by synthesizing ECM components, promoting collagen alignment and fibronectin unfolding, enhancing invasive behaviors of malignant and pre-malignant human breast cancer cells (108). This study provided a link between obesity and the increased myofibroblast populations observed in mammary adipose tissue, with the consequent increased ECM stiffness and tumor promotion.

However, there is also emerging evidence that CAAs influence tumor ECM remodeling. Adipocytes derived from human peri-prostatic adipose tissue primed by prostate carcinoma cells were found to upregulate TNF-α, osteopontin, and MMP9, which are known to regulate ECM architecture (109). Furthermore, adipocytes secrete and process collagen VI, which provides pro-survival signals at the early stages of tumor growth in murine mammary ductal carcinoma (also consistent with observations in human breast cancer tissues), and its cleavage product endotrophin, promotes mammary tumor growth via recruitment of endothelial cells and macrophages that subsequently stimulate angiogenesis, fibrosis and an inflammatory environment (110). CAA-derived endotrophin induced TGF-β mediated EMT in mammary cancer cells (111) and CAAs also promoted tumor cell invasiveness by upregulation of versican and leptin in renal cell carcinoma cell lines (112). Overall, these observations provide circumstantial evidence for a role for CAA in the microenvironment and particularly the formation of a tumor-permissive ECM, suggesting that more work using *in vivo* models is warranted.

### Tumor infiltrating lymphocytes (TILs)

Tumor infiltrating lymphocytes (TILs) which include CD8+ cytotoxic T lymphocytes (CTLs), CD4+ T helper lymphocytes (Th), CD4+ regulatory T lymphocytes (Treg), γδT cells, and B-cells. Tumor-suppressing roles of T helper and cytotoxic T cells have been widely studied (113, 114). However, TILs can also contribute to the tumor-promotion through the interplay with other stromal components, such as macrophages or neutrophils and the cytokines they secrete. In response to IL-23, IL-6, and TGF-β in the tumor microenvironment, γδT cells secrete IL-17 and induce angiogenesis in a transplantable sarcoma model in mice (115) and in response to tumor-derived IL-1β, they produce IL-17 and induce systemic, G-CSF-mediated activation of neutrophils in mammary tumors to promote cancer-cell metastasis to the lungs (116). It has also been observed that IL-4 secreting CD4+ T lymphocytes were able to indirectly promote tumor invasiveness and pulmonary metastasis of mammary tumors via enhancing pro-tumor properties of tumor associated macrophages (117).

Tregs, on the other hand, are thought to exert an immunosuppressive influence within the tumor microenvironment and are able to induce apoptosis of NK cells via direct cell-to-cell contact as well as through TGF-β secretion (118), but under some circumstances may promote tumor angiogenesis via the production of VEGFA, as has been demonstrated in an ovarian cancer murine xenograft model (119).

Activated B-cells contribute to pre-malignant inflammatory responses and to enhance tumor growth in the HPV-16-driven multistage epidermal carcinogenesis model (120). In castration resistant prostate cancer, tumor infiltrating B-cells secrete lymphotoxin (LT) α:β which engages with LTβR on cancer cells and activates the STAT3 pathway to promote androgen-independent cancer cell growth (121). Interestingly upon STAT3 activation in B-cells there has been observed an increased angiogenesis in B16 melanoma and Lewis lung cancer models, however a direct role of B-cells in angiogenesis is still unclear (122).

TILs have not been directly implicated in the production of ECM. Nevertheless, they are important regulators of the cellular composition of the tumor microenvironment and play indirect roles in the establishment of a tumor-promoting matrix via their role in ECM remodeling. Lymphocytes express ECM modifying enzymes such as MMPs and the urokinase plasminogen activator system in order to traverse basement membrane (123). It has been demonstrated that *ex vivo* purified peripheral lymphocytes respond to chemokine and cytokine stimulation by increased MMP-9 production (124). Furthermore, fibronectin-mediated activation of focal adhesion kinase (FAK) regulates the expression and release of MMP-2 and MMP-9 by T lymphocytes *in vitro* (125). T lymphocytes isolated from the spleens of mammary tumor-bearing mice exhibit elevated production of MMP-9 at both the mRNA and protein level (126). Besides MMP production human T-cells are capable of inducing MMP-9, MMP-1, and MMP-3 expression *ex vivo* in human endothelial cells through CD40/CD40 ligand interaction (127). Another study has demonstrated that lymphoma cells were able to induce MMP-9 expression in fibroblasts and macrophages (128). While the foregoing demonstrates that lymphocytes can produce ECM remodeling enzymes, there is as yet no evidence to suggest that this is a feature of tumor growth and progression *in vivo*. More work is therefore required to determine whether lymphocyte-mediated ECM remodeling has a direct function in tumor progression and cancer cell plasticity.

### Cancer cells

While much of the aberrant ECM production and remodeling in cancer is initiated within the stroma (129), cancer cells themselves can produce some ECM proteins. Proteomics-based analysis of xenografted breast cancers revealed that highly aggressive and metastatic cancer cell lines produced ECM components such as fibronectin, fibrinogen, laminins, periostin, collagens I, III, IV, V, and VI, transglutaminase 2, and hyaluronan. Of note, production of certain components of the ECM is associated with increased metastatic potential of cancer cells—particularly LTBP3, SNED1, EGLN1, and S100A2. LTBP3 has been previously implicated in the regulation of TGF-β secretion and promotion of tumor invasion and metastasis. S100A2 overexpression has been shown to promote lung metastasis of non-small-cell lung carcinoma cells (130, 131).

The best documented and arguably principal path to the ECM conditioning by cancer cells is through deregulation and/or increased production of ECM-modifying enzymes. Uncontrolled tumor cell proliferation and limited tissue blood supply induces intra-tumoral hypoxia, which in turn induces expression of the gene encoding the collagen and elastin cross-linking enzyme lysyl oxidase (LOX) in human tumor cells (132). LOX-mediated collagen and elastin crosslinking leads to stiffening of the ECM and enhances invasive migration of human breast and cervical cancer cells lines under hypoxic conditions (133). Furthermore, ECM stiffening activates integrin signaling, promote focal adhesion assembly and enhance PI3 Kinase (PI3K) activity that leads to tumor progression and invasion (20, 134). ECM stiffening also promotes growth, survival, migration, and proliferation of cancer cells via integrin ligation and engagement of the Rho-ROCK, PI3K, and MAP/ERK signaling pathways (135) and acute compressive stress such as that encountered in the microenvironment during early stages of epithelial tumor growth can activate Rho-ROCK signaling and downstream actomyosin tension to enhance proliferation and generate an EMT profile (136).

Interestingly, hypoxia also affects the ability of tumor cells to produce collagen-modifying enzymes [reviewed in (137)]. Hypoxia-mediated upregulation of collagen prolyl 4-hydroxylases (P4H) in breast cancer cells has been found to be an important contributor to cancer cell invasion and metastasis (138). Another collagen-modifying enzyme, procollagen-lysine 2-oxyglutarate 5-dioxygenase 2 (PLOD2), was implicated in fibrillar collagen formation by breast cancer cells and as a result enhanced breast cancer cell metastasis to lymph nodes and lung (139).

Another class of ECM-modifying enzymes produced by cancer cells are matrix metalloproteinases. It has been observed that hypoxia-induced upregulation of MMP2 and MMP9 in breast and colon cancer cells contributed to tumor cell invasion (140, 141) and a membrane-bound form of MMP—MT1-MMP (MMP-14) is also induced via hypoxia in breast and renal carcinoma cells (141). MMP14 is required for multicellular invasion of breast cancer cells (142) and is key to breakdown of the basement membrane prior to invasion (143). Induction of an EMT program in breast cancer cells causes MMP production, and increased expression of MMP3, MMP10, and MMP13 was observed upon TGF-β stimulation of human breast cancer cell lines. Upon induction of EMT via hydrogen peroxide treatment in murine mammary epithelial cells, production of MMP2, MMP12, and MMP13 was observed (144).

In another example, enforced activation of Rho kinase signaling in pancreatic ductal adenocarcinomas (PDAC) in mice caused increased production of Mmp10 and Mmp13, which were released in micro-vesicles. This enabled efficient collagen degradation within close vicinity of the cancer cells and as a result enhanced PDAC cell proliferation and collective invasion (145). These observations add to emerging evidence that tumor epithelial cells release micro-vesicles that induce extracellular matrix remodeling (146–148).

Actin-rich membrane structures such as focal adhesions and invadopodia have also been implicated in ECM remodeling by cancer cells. These structures contain an assembly of scaffolding proteins (WASP, N-WASP, and VASP) paired with actin-remodeling proteins (such as cortactin and gelsolin). These structures are able to incorporate integrin-mediated signaling and recruit Rho GTPases, myosins, Src kinases, and dynamin (149). Focal adhesions and invadopodia are essential for cell migratory behavior *in vitro* and for actomyosin-based contractility (150).

Focal adhesions are known to integrate multiple signaling inputs and transduce them across the cell (151). However, a recent study showed that cancer cells are also able to degrade the ECM at focal adhesion sites via recruitment of MMP14 (152). An important characteristic of migrating cancer cells is the formation of actin-rich membrane extensions termed invadopodia. In cancer cells, mature invadopodia are enriched in MMP2, MMP9, and MMP14. It is important to note that MMP2 and 9 are particularly essential for type IV collagen remodeling and subsequent breaching of the basement membrane (153). Invadopodia are also important for the extravasation of squamous carcinoma, breast cancer, and bladder cancer cells as well as melanoma cells (154). Whilst *in vitro* studies have shown that invadopodia formation is important for basement membrane penetration, conclusive *in vivo* evidence is lacking regarding the physiological roles of invadopodia.

In this section and summarized in Table 1, we have provided snapshots of the biology of the many cell types and discussed what is known about how they influence tumor cell plasticity in the context of ECM, to regulate EMT and stemness. In Section Molecular Regulators of the ECM That Influence Cancer Cell Plasticity below, we will discuss the molecular regulators that are employed across these various cell types to carry out cellular processes and promote tumor progression.

**Table 1:** Cellular regulators of the ECM and cancer cell plasticity.

**Cell type**	**ECM changes**	**Influence on cancer cells and their plasticity**	**References**
Cancer associated fibroblasts (CAFs)	Collagen production, fiber alignment and increased ECM stiffness	Growth and motility, invasion, angiogenesis, increased metastatic potency	(26, 29, 41, 43, 155)
	Establishing actomyosin tracks	Migration, invasion	(44, 156)
	MMP-1 secretion	Motility, invasiveness	(157)
			
	MMP-2 and MMP-9 secretion	EMT	(33)
	Tenascin C production	Invasiveness	(42)
			
	Periostin production	Stem cell niche maintenance	(36)
	Production of collagens, fibronectin, osteopontin and periostin leading to desmoplasia	Proliferation	(64, 65)
Tumor associated macrophages (TAMs)	Production of MMPs (1, 9, 12 and 14), serine proteases, cathepsins (B, S, C, L, Z), lysosomal enzymes and ADAMs	Invasion	(64, 66, 158)
	Secretion of ECM remodeling enzymes and liberation of ECM-bound growth factors	Proliferation, motility	(64, 68)
Tumor associated neutrophils (TANs)	Secretion of MMP9	Proliferation, invasiveness, angiogenesis, extravasation, metastasis	(82, 83)
	Elastase production	EMT	(84)
	NETosis, upregulation of MMP9, cathepsin G and neutrophil elastase	Proliferation, migration and angiogenesis	(91)
	Establishment of collagen tracks	Invasion	(87)
Cancer associated adipocytes (CAAs)	Secretion of MMP9 and osteopontin	proliferation, motility	(109)
	Production and processing of collagen VI	Survival, growth, angiogenesis, EMT	(110, 111)
	Secretion of versican	Invasion, progression	(112)
Cancer cells (CSs)	Secretion of LOX that crosslinks collagen and elastin, increasing ECM stiffness	Proliferation, survival, invasion	(132, 133)
	Secretion of ECM-modifying enzymes: collagen prolyl 4-hydroxylases (P4H), procollagen-lysine 2-oxyglutarate 5-dioxygenase 2 (PLOD2)	Invasion, metastasis	(138, 139)
	MMP2 and MMP9, Mmp10 and Mmp13, Mmp14 secretion and expression leading to collagen remodeling	Invasion, proliferation, cell migration, collective invasion	(140, 141, 145)

## Molecular regulators of the ECM that influence cancer cell plasticity

The microenvironment is precisely regulated by several molecular players that have evolved to return this system to its steady state in the shortest possible time following perturbation, while also permitting it to adapt quickly to changed circumstances such as injury or disease. This ability to quickly adapt to circumstances and resilience under injury can be co-opted by disease states such as cancer and accounts for a significant component of the plasticity exhibited by cancer cells. Changes in the mechanical and biochemical properties of the ECM have been linked to cancer cell plasticity that promotes increased invasiveness and metastatic potential (159). Furthermore, tumor cells that have undergone EMT, TAMs, TANs, and CAFs are all capable of producing ECM components and degrading and remodeling the ECM to facilitate tumor cell plasticity and disease progression as we have discussed above. Below, we discuss some of the molecular players that mediate ECM production and re-modeling by these cell types, to promote tumor progression and cancer cell plasticity.

Tumor and stromal cells employ several signaling pathways that regulate the biochemical and biomechanical interactions between the parenchyma and the microenvironment to establish, remodel and maintain the ECM. Whereas normal epithelial cells produce only small amounts of ECM, fibroblasts, tumor cells, and certain immune cells like macrophages have the capacity to produce vast quantities of the proteins that form this meshwork scaffold and are largely responsible for its production and maintenance. Nevertheless, they do not perform this task independently, but are regulated by biochemical and biomechanical cues from the parenchyma.

Established molecular pathways that regulate ECM properties include TGF-β, CTGF, and Wnt signaling axes and the mediators of the YAP signaling system, which are discussed here.

### TGF-β signaling

TGF-β family members are multifunctional cytokines with roles in wound healing, tissue repair, and cancer, and regulate a signaling cascade largely involved in the transcriptional regulation of genes that control EMT and stemness (160). Activation of this signaling cascade is initiated by the binding of a TGF-β ligand to a Type II receptor serine/threonine kinase on the cell surface, resulting in recruitment of the type I receptor to the complex. The Type II receptor trans-phosphorylates the Type I receptor at serine and threonine residues in the highly conserved juxta-membrane GS domain, and the phosphorylated Type I receptor propagates signaling by phosphorylating the SMAD signal transducer proteins. SMAD proteins are latent transcription factors and once phosphorylated can translocate into the nucleus and regulate transcription of target genes in cooperation with nuclear cofactors and the transcription machinery (161).

The numerous TGF-β superfamily of ligands (at least 42 in humans, 9 in fly, and 6 in worm) comprise two major subfamilies based on structure and function. These are the TGF-β/Activin/Nodal subfamily and the bone morphogenetic protein/growth and differentiation factor/Müllerian inhibiting substance subfamily (BMP/GDF/MIS) (162). While each of these cytokines can elicit a different set of responses via the TGF-β signaling pathway underlying the highly pleiotropic nature of this pathway, they share several common features of sequence, structure and function, namely, six conserved cysteine residues which generate a cysteine knot structure via three disulfide bonds (163) and the ability to act only in the dimerized form. Furthermore, there are 7 type I and 5 type II receptor Ser/Thr kinases in humans. Both receptor types have an N-terminal ligand-binding domain, a transmembrane domain and a C-terminal Ser/Thr kinase domain. Type I (but not type II) receptors also contain a characteristic SGSGSG sequence, the “GS domain,” which is phosphorylated by the Type II receptor. SMAD proteins are divided into three functional classes: the receptor-regulated SMADs (R-SMADs), the co-mediator SMAD (Co-SMAD), and the inhibitory SMADs (I-SMADs). R-SMADs are directly serine-phosphorylated at a conserved C-terminal SSXS motif by the Type I receptor. R-SMADs comprise the BMP-receptor-regulated SMADs (1, 5, and 8), and the TGF-β/Activin receptor-regulated SMADs (2 and 3). The Co-SMAD, SMAD4, can hetero-dimerize with phosphorylated R-SMADs and is involved in mediating their translocation into the nucleus. I-SMADs (6 and 7) negatively regulate signaling by competing with R-SMADs for receptor and Co-SMAD binding. They are also able to target receptors for degradation, thereby regulating signal flux through this pathway. Therefore, the high level of redundancy present within this signaling pathway has the potential to greatly influence context-specific outcomes mediated by the activation of diverse and distinct transcriptional profiles.

TGF-β is secreted by many cell types, including those abundant within the tumor microenvironment such as activated macrophages (164), endothelial cells (165), and fibroblasts (166). Tumor cells also secrete TGF-β, which can elicit context-dependent responses that suppress tumor growth at early stages of the disease, but promote tumor progression at later stages (167). Nevertheless, two key functions of TGF-β signaling in the cancer microenvironment are regulation of immune evasion and ECM remodeling. TGF-β signaling has been demonstrated to regulate phenotypic plasticity of cancer cells arising in diverse tissues including the skin (168), intestine (169), breast (170), and lung (171).

TGF-β signaling controls the transcription of a suite of genes, including those encoding ECM proteins such as collagen, and ECM remodeling enzymes such as lysyl oxidase (172, 173), via regulation of the transcription factor MYC. This signaling pathway is therefore associated with increased ECM stiffness, which induces proliferation and mesenchymal behavior in resident tumor cells by promoting integrin ligation and downstream signaling pathways. Interestingly, local concentrations of TGF-β are enhanced and its spatial activity regulated by its immobilization onto the ECM (174), which results in the capacity to influence cancer cell plasticity at specific regions of the tumor.

Given the high level of reciprocal crosstalk between TGF-β, the ECM and cellular plasticity as detailed above, this signaling pathway is well-established as a key target in cancer therapy. However, the pleiotropic and context-dependent functions of the pathway have hampered the development of tractable agents that reliably target TGF-β-regulated tumor cell plasticity.

### CTGF signaling

The Connective Tissue Growth Factor (CTGF, sometimes referred to as CCN2) is a member of the CCN family of non-structural ECM proteins and is therefore most appropriately termed a matricellular protein. It can interact with a large array of signaling molecules, including bone morphogenetic proteins (BMP), TGF-β, VEGF, IGF, and Wnt ligands as well as directly bind trans-membrane receptors such as integrins, Notch receptors, TGF-β receptors, and lipoprotein receptor-related proteins (LRPs) to elicit the corresponding signaling cascades (175, 176). In cancer, a key function of CTGF is to mediate fibronectin production by stromal cells downstream of TGF-β signaling, which transcriptionally regulates *CTGF*. Fibronectin in turn determines the biosynthesis and fibrillogenesis of collagen 1, the main component of the cancer ECM.

CTGF has been shown to regulate the MET of head and neck cancer cells (177) and drug resistance in glioblastoma (178), both via a mechanism involving the re-expression of pluripotency genes. Furthermore, CTGF inhibition reduces the growth of metastatic melanoma in an animal model (179). These data suggest that CTGF plays a role in the metastatic colonization by cancers of distal sites by enhancing pluripotency and MET. It is not clear whether these two functions are linked and to what extent they are also mediated by crosstalk with other, closely regulated, CCN family proteins.

### Wnt/β-catenin signaling

Wnt ligands are a large family of secreted glycoproteins that can activate three distinct intracellular signaling pathways—the β-catenin pathway (also known as the canonical Wnt signaling pathway), the planar cell polarity pathway (involving Jun N-terminal kinase [JNK]-mediated cytoskeleton rearrangements) and the Wnt/Ca^2+^ pathway, by interacting with cell surface bound Frizzled receptors. Critical to Wnt signaling are low density lipoprotein (LDL) receptor-related proteins, which act as co-receptors of the Wnt signal (180). Transduction of the signal via Frizzled is mediated by the intracellular protein Disheveled (181), which acts to inhibit the kinase GSK3B through its interaction with Axin (182–184).

GSK3B exists in a large multi-protein complex containing Axin, β-catenin, and the adenomatous polyposis coli (APC) protein (185–188). In the absence of Wnt ligands, β-catenin is maintained in the phosphorylated state at its amino-terminal Ser/Thr residues by GSK3B. Phosphorylated β-catenin is ubiquitinated by β-TRCP and thereby targeted for degradation via the proteasome pathway (189, 190). Wnt ligand binding to Frizzled receptors causes GSK3B inactivation by Disheveled, resulting in the accumulation of non-phosphorylated β-catenin, which cannot be ubiquitinated and is therefore protected from proteasome mediated degradation. β-catenin associates with the T cell factor/lymphocyte enhancer factor (Tcf/LEF) family of transcription factors and functions as a co-activator of transcription upon translocation of the β-catenin/Tcf/LEF complex to the nucleus (191). In the absence of Wnt ligand, non-phosphorylated β-catenin levels are low and Tcf proteins are bound to various inhibitory molecules (including Groucho proteins, CtBP, and in Drosophila, CBP), preventing the transcription of target genes (192–195).

The role of Wnt/β-catenin signaling in EMT has been well known for some time. Wnt signaling was first demonstrated to stabilize and thereby control the turnover of the EMT regulator Snail1 (196) and increase the expression of two further EMT regulators, Slug (197) and Twist (198). *In vivo* evidence for the role of Wnt signaling in regulating EMT and metastasis has been provided in breast cancer (199) and pancreatic cancer (200). It is also becoming clear that that non-canonical Wnt signaling initiated by Wnt5b regulates metastasis via EMT (201).

The Wnt signaling pathway has also been demonstrated as a regulator of stemness, both in stem cell maintenance and renewal, for example in the intestine (202), as well as in stem cell differentiation and fate determination via transcriptional targets such as Sox9 (203). This can be brought about by the activation of distinct subsets of transcriptional targets and by signaling crosstalk between this signaling pathway and others. For instance, crosstalk between prostaglandin signaling and Wnt signaling is required for the developmental specification of stem cell populations in the hematopoietic system as well as in the liver and other organs (204), and also in the de-differentiation process that gives rise to stem-like cells in cancers such as cutaneous squamous cell carcinoma (20). Taken together, these observations firmly place the Wnt/β-catenin signaling pathway as a key regulator of cell plasticity in normal development, but also in cancer.

Functional interactions between the Wnt signaling pathway and the extracellular matrix are being uncovered, most prominently in normal development of bone, where it is emerging that mechanotransduction signaling initiated by ECM stiffness regulates Wnt secretion (205). These observations have obvious implications for the cancer context in which enhanced mechanotransduction is a well-established pathology.

### Rho/ROCK signaling

The 22-member RHO family of small GTPases are named for their homology to the Ras proto-oncogenes. Of these, the best characterized are RHOA, RAC1, and CDC42, which have distinct roles in regulating actin polymerization and turnover, and myosin contractility (206). These small GTPases are co-opted by many signaling pathways to modify the actomyosin cytoskeleton and thereby underpin most cellular processes. ROCK kinases 1 and 2 (207) are key effectors of signaling through RHOA and are activated by direct binding of GTP-bound active RHOA (208–210). Active ROCK kinases signal via a collection of context-dependent downstream pathways that are mainly involved in regulation of actomyosin cytoskeleton properties including actin polymerization and cytoskeletal contractility. Key mediators of ROCK signaling are the LIM kinases, whose major role is to phosphorylate and inhibit the actin severing Cofilins, thereby stabilizing the actin cytoskeleton and promoting invasiveness through generation of a tumor-permissive network (211). LIMK2 has also been shown to integrate RHO signaling and p53 functions, thereby mediating cell survival functions in cancer cells, with implications for tumor plasticity and progression (212). Signaling downstream of ROCK also regulates myosin contractility via ROCK-mediated phosphorylation and activation of the regulatory myosin light chain MLC2, as well as phosphorylation and inactivation of the myosin binding subunit of the myosin phosphatase MYPT1 (213). These two signaling arms, resulting in actin cytoskeleton stabilization and myosin contractility, therefore have a major role in regulating intracellular tension and thereby integrate several mechanotransduction pathways within the cell, including the Wnt and YAP pathways.

A key role for RHO-ROCK signaling has been delineated in the tissue and tumor microenvironments, to complement its well-established function in cell migration and adhesion (214). The pathway accomplishes this via the increased production of ECM components to balance intracellular tension, thereby maintaining mechano-reciprocity (20). This recent appreciation that ROCK activity regulates ECM production and remodeling [reviewed in (135)] highlighted the possibility of novel negative regulators of this pathway that may be of therapeutic utility. Accordingly, 14-3-3ζ, which belongs to the 14-3-3 family of molecular adaptors and chaperones, has been shown to bind to and promote the activity of the myosin phosphatase targeting subunit Mypt1, thereby increasing signaling flux through the RHO-ROCK pathway (23). Interestingly, a family of sphingosine mimetics, which had been previously demonstrated to inhibit 14-3-3 by disrupting dimer formation (215), accelerates the production of ECM components required to normalize the cutaneous microenvironment thereby hastening wound healing. However, this mechanism is hijacked by cancers such as cutaneous squamous cell carcinoma, where 14-3-3ζ is downregulated frequently and is associated with tumor progression (23). These observations suggest that as in the case of M2 polarized “wound healing type” macrophages, cancers can exploit mechanisms that have evolved to facilitate wound healing, to assist with tumor progression. Further work is required to determine whether this phenomenon may be exploited in cancer therapy or indeed whether other mechanisms mediating ECM re-establishment may be similarly engaged to target the tumor microenvironment as a novel therapy approach.

### Hippo signaling

The still rather enigmatic Salvador/Warts/Hippo pathway is a highly conserved signaling pathway and acts as a controller of organ size in animals by regulating the balance between cell proliferation and death (216). The pathway has evolved to control the activity of the transcriptional regulators YAP and its paralog TAZ, which promote proliferation by associating with the TEAD transcription factors (217). In its activated state, the pathway consists of a Ser/Thr kinase cascade initiated by the transmembrane cadherin FAT that results in the phosphorylation of YAP/TAZ to create a binding site for 14-3-3 proteins. Upon binding of 14-3-3, YAP/TAZ is sequestered in the cytoplasm and is therefore not available in the nucleus to induce the transcription of target genes. The YAP/TAZ inhibitory kinase cascade is regulated by a variety of different inputs, including hormone and growth factor signaling. However, YAP has also been reported to be regulated by RHO GTPase activity mediated by ROCK, in a Hippo pathway-independent mechanism (218) and thereby links mechanotransduction to the transcription of genes that promote cell proliferation (219). More recently, it has been revealed that TEAD2 regulates the expression of EMT genes by directly controlling the sub-cellular localization of YAP/TAZ (220). As such, it is a key mediator of cancer cell plasticity and neoplastic progression downstream of changes in ECM stiffness. The Hippo signaling pathway has also demonstrated to engage in crosstalk with the Wnt/β-catenin signaling pathway and Notch pathway in the context of hepatocellular cancers (221), suggesting that the three mechanotransduction pathways are capable of cooperating to promote tumor progression via the regulation of plasticity, suggesting that Hippo signaling could contribute to the mechano-reciprocal feed forward loop that we have previously proposed (135).

These and other signaling molecules that regulate ECM structure and function to influence cancer cell plasticity both directly and indirectly are summarized in Table 2.

**Table 2:** Molecular regulators of the ECM and cancer cell plasticity.

**Pathway**	**Effects on the ECM**	**Plasticity-dependent cellular processes influenced**	**References**
TGF-β	Upregulation of collagen, lysil oxydase expression in cancer cells and stiffening of ECM	Motility and proliferation	(172, 173)
	Upregulation of tenascin C in CAFs	Invasiveness	(42)
Rho/ROCK	Remodeling of focal adhesions	Cell migration and adhesion	(214)
	Activation in tumor epithelial cells induces production of collagen, fibronectin, tenascin C, periostin by fibroblasts, increases ECM stiffness	Tumor progression, enhanced wound healing	(20, 23)
Notch	Indirect - influencing ECM sensing by integrin; maintenance of stemness	Normal stem cell maintenance; acquisition of CSC phenotype	(222, 223)
FGF	Influences hedgehog-induced ECM production by CAFs; cooperates with TGF-β in EMT	Acquisition of stem cell phenotype; EMT	(37, 224)
HGF	Mediates fibroblast-tumor cell communication; indirectly facilitates ECM degradation	EMT	(225)

## Implications for cancer therapy

As we have discussed above, cancer cell plasticity permits tumors to adopt shifting identities that allow them to adapt to changing environments, modify their microenvironment to suit their needs and evade the immune system. In this effort, cancers can co-opt and deftly commandeer many of the body's own normal homeostatic processes such as wound healing, immune surveillance and maintenance of the stem cell niche. While this poses a significant challenge to cancer therapy, it also provides us with an opportunity to target the aberrant microenvironment that has been built around the tumor. A key vulnerability of tumors exhibiting plasticity is their need to subvert the activities of genetically normal stromal cells for their own purposes by biochemical and biomechanical means. This provides us with an opportunity to block signals traveling between cancers and their stroma pharmacologically, using antibody therapy or by modifying the mechanical environment of the tumors; or indeed a combination of all three. Coupled with precision therapies tailored to the tumor genotype, a multi-pronged approach targeting the tumor as well as its microenvironment has the potential to revolutionize cancer therapy.

As a note of caution however, it is important to appreciate that plasticity may also provide tumors with the means to evade such combination therapies. It is therefore imperative that the core set of principles driving cancer cell plasticity be soundly researched and fully appreciated. Given the plethora of autochthonous animal models of human cancers and more recently the patient-derived xenograft models being propagated in immunologically humanized animals, we believe the tools are being rapidly assembled to make this a reality.

## Author contributions

VP wrote the review and edited the manuscript. MK wrote the review and edited the manuscript. SP wrote the review and edited the manuscript. MS conceived of the review, planned the outline, wrote the review and edited the manuscript.

### Conflict of interest statement

The authors declare that the research was conducted in the absence of any commercial or financial relationships that could be construed as a potential conflict of interest.

## References

[1] Bacac M Stamenkovic I. Metastatic cancer cell. Annu Rev Pathol. (2008) 3:221–47. 10.1146/annurev.pathmechdis.3.121806.151523 18233952

[2] Quail DF Joyce JA. Microenvironmental regulation of tumor progression and metastasis. Nat Med. (2013) 19:1423–37. 10.1038/nm.3394 24202395 PMC3954707

[3] Tsai JH Yang J. Epithelial-mesenchymal plasticity in carcinoma metastasis. Genes Dev. (2013) 27:2192–206. 10.1101/gad.225334.113 24142872 PMC3814640

[4] Orkin SH Zon LI. Hematopoiesis: an evolving paradigm for stem cell biology. Cell (2008) 132:631–44. 10.1016/j.cell.2008.01.025 18295580 PMC2628169

[5] Marusyk A Tabassum DP Altrock PM Almendro V Michor F Polyak K. Non-cell-autonomous driving of tumour growth supports sub-clonal heterogeneity. Nature (2014) 514:54–8. 10.1038/nature13556 25079331 PMC4184961

[6] Nassar D Blanpain C. Cancer stem cells: basic concepts and therapeutic implications. Annu Rev Pathol. (2016) 11:47–76. 10.1146/annurev-pathol-012615-044438 27193450

[7] Batlle E Clevers H. Cancer stem cells revisited. Nat Med. (2017) 23:1124–34. 10.1038/nm.4409 28985214

[8] Villa-Diaz LG Kim JK Laperle A Palecek SP Krebsbach PH. Inhibition of focal adhesion kinase signaling by integrin alpha6beta1 supports human pluripotent stem cell self-renewal. Stem Cells (2016) 34:1753–64. 10.1002/stem.2349 26930028

[9] Cattavarayane S Palovuori R Tanjore Ramanathan J Manninen A. alpha6beta1- and alphaV-integrins are required for long-term self-renewal of murine embryonic stem cells in the absence of LIF. BMC Cell Biol. (2015) 16:3. 10.1186/s12860-015-0051-y 25886986 PMC4348401

[10] Popov C Radic T Haasters F Prall WC Aszodi A Gullberg D. Integrins alpha2beta1 and alpha11beta1 regulate the survival of mesenchymal stem cells on collagen I. Cell Death Dis. (2011) 2:e186. 10.1038/cddis.2011.71 21796158 PMC3199721

[11] Plaks V Kong N Werb Z. The cancer stem cell niche: how essential is the niche in regulating stemness of tumor cells? Cell Stem Cell (2015) 16:225–38. 10.1016/j.stem.2015.02.015 25748930 PMC4355577

[12] Kumar S Weaver VM. Mechanics, malignancy, and metastasis: the force journey of a tumor cell. Cancer Metastasis Rev. (2009) 28:113–27. 10.1007/s10555-008-9173-4 19153673 PMC2658728

[13] Malik R Lelkes PI Cukierman E. Biomechanical and biochemical remodeling of stromal extracellular matrix in cancer. Trends Biotechnol. (2015) 33:230–6. 10.1016/j.tibtech.2015.01.004 25708906 PMC4380578

[14] Venning FA Wullkopf L Erler JT. Targeting ECM disrupts cancer progression. Front Oncol. (2015) 5:224. 10.3389/fonc.2015.00224 26539408 PMC4611145

[15] El-Haibi CP Bell GW Zhang J Collmann AY Wood D Scherber CM. Critical role for lysyl oxidase in mesenchymal stem cell-driven breast cancer malignancy. Proc Natl Acad Sci USA. (2012) 109:17460–5. 10.1073/pnas.1206653109 23033492 PMC3491529

[16] Singleton PA. Hyaluronan regulation of endothelial barrier function in cancer. Adv Cancer Res. (2014) 123:191–209. 10.1016/B978-0-12-800092-2.00007-1 25081530 PMC4470488

[17] Nikitovic D Tzardi M Berdiaki A Tsatsakis A Tzanakakis GN. Cancer microenvironment and inflammation: role of hyaluronan. Front Immunol. (2015) 6:169. 10.3389/fimmu.2015.00169 25926834 PMC4396412

[18] Alexander S Koehl GE Hirschberg M Geissler EK Friedl P. Dynamic imaging of cancer growth and invasion: a modified skin-fold chamber model. Histochem Cell Biol. (2008) 130:1147–54. 10.1007/s00418-008-0529-1 18987875

[19] Egeblad M Rasch MG Weaver VM. Dynamic interplay between the collagen scaffold and tumor evolution. Curr Opin Cell Biol. (2010) 22:697–706. 10.1016/j.ceb.2010.08.015 20822891 PMC2948601

[20] Samuel MS Lopez JI Mcghee EJ Croft DR Strachan D Timpson P. Actomyosin-mediated cellular tension drives increased tissue stiffness and beta-catenin activation to induce epidermal hyperplasia and tumor growth. Cancer Cell (2011) 19:776–91. 10.1016/j.ccr.2011.05.008 21665151 PMC3115541

[21] Guo W Giancotti FG. Integrin signalling during tumour progression. Nat Rev Mol Cell Biol. (2004) 5:816–26. 10.1038/nrm1490 15459662

[22] Bainbridge P Wound healing and the role of fibroblasts. J Wound Care (2013) 22:407–8, 410–12. 10.12968/jowc.2013.22.8.407 23924840

[23] Kular J Scheer KG Pyne NT Allam AH Pollard AN Magenau A. A negative regulatory mechanism involving 14–3-3zeta limits signaling downstream of ROCK to regulate tissue stiffness in epidermal homeostasis. Dev Cell (2015) 35:759–74. 10.1016/j.devcel.2015.11.026 26702834

[24] Trapani V Bonaldo P Corallo D. Role of the ECM in notochord formation, function and disease. J Cell Sci. (2017) 130:3203–11. 10.1242/jcs.175950 28883093

[25] Nagy N Barad C Hotta R Bhave S Arciero E Dora D. Collagen 18 and agrin are secreted by neural crest cells to remodel their microenvironment and regulate their migration during enteric nervous system development. Development (2018) 145. 10.1242/dev.160317 29678817 PMC5992596

[26] Ishii G Ochiai A Neri S. Phenotypic and functional heterogeneity of cancer-associated fibroblast within the tumor microenvironment. Adv Drug Deliv Rev. (2016) 99:186–96. 10.1016/j.addr.2015.07.007 26278673

[27] Kalluri R. The biology and function of fibroblasts in cancer. Nat Rev Cancer (2016) 16:582–98. 10.1038/nrc.2016.73 27550820

[28] Dvorak HF Tumors: wounds that do not heal. Similarities between tumor stroma generation and wound healing. N Engl J Med. (1986) 315:1650–9. 10.1056/NEJM198612253152606 3537791

[29] Coussens LM Werb Z. Inflammation and cancer. Nature (2002) 420:860–7. 10.1038/nature01322 12490959 PMC2803035

[30] Augsten M. Cancer-associated fibroblasts as another polarized cell type of the tumor microenvironment. Front Oncol. (2014) 4:62. 10.3389/fonc.2014.00062 24734219 PMC3973916

[31] Yu Y Xiao CH Tan LD Wang QS Li XQ Feng YM. Cancer-associated fibroblasts induce epithelial-mesenchymal transition of breast cancer cells through paracrine TGF-beta signalling. Br J Cancer (2014) 110:724–32. 10.1038/bjc.2013.768 24335925 PMC3915130

[32] Fiaschi T Giannoni E Taddei ML Cirri P Marini A Pintus G. Carbonic anhydrase IX from cancer-associated fibroblasts drives epithelial-mesenchymal transition in prostate carcinoma cells. Cell Cycle (2013) 12:1791–801. 10.4161/cc.24902 23656776 PMC3713137

[33] Giannoni E Bianchini F Masieri L Serni S Torre E Calorini L. Reciprocal activation of prostate cancer cells and cancer-associated fibroblasts stimulates epithelial-mesenchymal transition and cancer stemness. Cancer Res. (2010) 70:6945–56. 10.1158/0008-5472.CAN-10-0785 20699369

[34] Gattazzo F Urciuolo A Bonaldo P. Extracellular matrix: a dynamic microenvironment for stem cell niche. Biochim Biophys Acta (2014) 1840:2506–19. 10.1016/j.bbagen.2014.01.010 24418517 PMC4081568

[35] Meran L Baulies A Li VSW. Intestinal stem cell Niche: the extracellular matrix and cellular components. Stem Cells Int (2017) 2017:7970385. 10.1155/2017/7970385 28835755 PMC5556610

[36] Malanchi I Santamaria-Martinez A Susanto E Peng H Lehr HA Delaloye JF. Interactions between cancer stem cells and their niche govern metastatic colonization. Nature (2012) 481:85–U95. 10.1038/nature10694 22158103

[37] Cazet AS Hui MN Elsworth BL Wu SZ Roden D Chan CL. Targeting stromal remodeling and cancer stem cell plasticity overcomes chemoresistance in triple negative breast cancer. Nat Commun. (2018) 9:2897. 10.1038/s41467-018-05220-6 30042390 PMC6057940

[38] Shimoda M Principe S Jackson HW Luga V Fang H Molyneux SD. Loss of the Timp gene family is sufficient for the acquisition of the CAF-like cell state. Nat Cell Biol. (2014) 16:889–901. 10.1038/ncb3021 25150980

[39] Vermeulen L Melo FDSE Van Der Heijden M Cameron K De Jong JH Borovski T. Wnt activity defines colon cancer stem cells and is regulated by the microenvironment. Nat Cell Biol. (2010) 12:468–U121. 10.1038/ncb2048 20418870

[40] Chen WJ Ho CC Chang YL Chen HY Lin CA Ling TY. Cancer-associated fibroblasts regulate the plasticity of lung cancer stemness via paracrine signalling. Nat Commun. (2014) 5:3472. 10.1038/ncomms4472 24668028

[41] Van Helvert S Storm C Friedl P. Mechanoreciprocity in cell migration. Nat Cell Biol. (2018) 20:8–20. 10.1038/s41556-017-0012-0 29269951 PMC5943039

[42] De Wever O Nguyen QD Van Hoorde L Bracke M Bruyneel E Gespach C. Tenascin-C and SF/HGF produced by myofibroblasts *in vitro* provide convergent proinvasive signals to human colon cancer cells through RhoA and Rac. FASEB J. (2004) 18:1016–8. 10.1096/fj.03-1110fje 15059978

[43] Calvo F Ege N Grande-Garcia A Hooper S Jenkins RP Chaudhry SI. Mechanotransduction and YAP-dependent matrix remodelling is required for the generation and maintenance of cancer-associated fibroblasts. Nat Cell Biol. (2013) 15:637–46. 10.1038/ncb2756 23708000 PMC3836234

[44] Sanz-Moreno V Gaggioli C Yeo M Albrengues J Wallberg F Viros A. ROCK and JAK1 signaling cooperate to control actomyosin contractility in tumor cells and stroma. Cancer Cell (2011) 20:229–45. 10.1016/j.ccr.2011.06.018 21840487

[45] Qian BZ Pollard JW. Macrophage diversity enhances tumor progression and metastasis. Cell (2010) 141:39–51. 10.1016/j.cell.2010.03.014 20371344 PMC4994190

[46] Wynn TA Chawla A Pollard JW. Macrophage biology in development, homeostasis and disease. Nature (2013) 496:445–55. 10.1038/nature12034 23619691 PMC3725458

[47] Pollard JW. Trophic macrophages in development and disease. Nat Rev Immunol. (2009) 9:259–70. 10.1038/nri2528 19282852 PMC3648866

[48] Sica A Mantovani A. Macrophage plasticity and polarization: *in vivo* veritas. J Clin Invest (2012) 122:787–95. 10.1172/JCI59643 22378047 PMC3287223

[49] Xue J Schmidt SV Sander J Draffehn A Krebs W Quester I. Transcriptome-based network analysis reveals a spectrum model of human macrophage activation. Immunity (2014) 40:274–88. 10.1016/j.immuni.2014.01.006 24530056 PMC3991396

[50] Aras S Zaidi MR. TAMeless traitors: macrophages in cancer progression and metastasis. Br J Cancer (2017) 117:1583–91. 10.1038/bjc.2017.356 29065107 PMC5729447

[51] Wyckoff J Wang WG Lin EY Wang YR Pixley F Stanley ER. A paracrine loop between tumor cells and macrophages is required for tumor cell migration in mammary tumors. Cancer Res. (2004) 64:7022–9. 10.1158/0008-5472.CAN-04-1449 15466195

[52] Murdoch C Tazzyman S Webster S Lewis CE. Expression of Tie-2 by human monocytes and their responses to angiopoietin-2. J Immunol. (2007) 178:7405–11. 10.4049/jimmunol.178.11.7405 17513791

[53] Qian BZ Li J Zhang H Kitamura T Zhang J Campion LR. CCL2 recruits inflammatory monocytes to facilitate breast-tumour metastasis. Nature (2011) 475:222–5. 10.1038/nature10138 21654748 PMC3208506

[54] Dwyer AR Greenland EL Pixley FJ. Promotion of tumor invasion by tumor-associated macrophages: the role of CSF-1-activated phosphatidylinositol 3 kinase and Src family kinase motility signaling. Cancers (Basel) (2017) 9:E68. 10.3390/cancers9060068 28629162 PMC5483887

[55] Boyle ST Faulkner JW Mccoll SR Kochetkova M. The chemokine receptor CCR6 facilitates the onset of mammary neoplasia in the MMTV-PyMT mouse model via recruitment of tumor-promoting macrophages. Mol Cancer (2015) 14:115. 10.1186/s12943-015-0394-1 26047945 PMC4464622

[56] Bohrer LR Schwertfeger KL. Macrophages promote fibroblast growth factor receptor-driven tumor cell migration and invasion in a CXCR2-dependent manner. Mol Cancer Res. (2012) 10:1294–305. 10.1158/1541-7786.MCR-12-0275 22893608 PMC3553584

[57] Baghel KS Tewari BN Shrivastava R Malik SA Lone MUD Jain NK Macrophages promote matrix protrusive and invasive function of breast cancer cells via MIP-1 beta dependent upregulation of MYO3A gene in breast cancer cells. Oncoimmunology (2016) 5. 10.1080/2162402X.2016.1196299 PMC5006911 27622050

[58] Lin EY Pollard JW. Tumor-associated macrophages press the angiogenic switch in breast cancer. Cancer Res. (2007) 67:5064–6. 10.1158/0008-5472.CAN-07-0912 17545580

[59] Goswami S Sahai E Wyckoff JB Cammer M Cox D Pixley FJ. Macrophages promote the invasion of breast carcinoma cells via a colony-stimulating factor-1/epidermal growth factor paracrine loop. Cancer Res. (2005) 65:5278–83. 10.1158/0008-5472.CAN-04-1853 15958574

[60] Linde N Lederle W Depner S Van Rooijen N Gutschalk CM Mueller MM. Vascular endothelial growth factor-induced skin carcinogenesis depends on recruitment and alternative activation of macrophages. J Pathol. (2012) 227:17–28. 10.1002/path.3989 22262122

[61] Sica A Saccani A Bottazzi B Polentarutti N Vecchi A Van Damme J. Autocrine production of IL-10 mediates defective IL-12 production and NF-kappa B activation in tumor-associated macrophages. J Immunol. (2000) 164:762–7. 10.4049/jimmunol.164.2.762 10623821

[62] Bonde AK Tischler V Kumar S Soltermann A Schwendener RA. Intratumoral macrophages contribute to epithelial-mesenchymal transition in solid tumors. BMC Cancer (2012) 12:35. 10.1186/1471-2407-12-35 22273460 PMC3314544

[63] Wyckoff JB Wang Y Lin EY Li JF Goswami S Stanley ER. Direct visualization of macrophage-assisted tumor cell intravasation in mammary tumors. Cancer Res. (2007) 67:2649–56. 10.1158/0008-5472.CAN-06-1823 17363585

[64] Liguori M Solinas G Germano G Mantovani A Allavena P. Tumor-associated macrophages as incessant builders and destroyers of the cancer stroma. Cancers (Basel) (2011) 3:3740–61. 10.3390/cancers3043740 24213109 PMC3763394

[65] Afik R Zigmond E Vugman M Klepfish M Shimshoni E Pasmanik-Chor M. Tumor macrophages are pivotal constructors of tumor collagenous matrix. J Exp Med. (2016) 213:2315–31. 10.1084/jem.20151193 27697834 PMC5068227

[66] Condeelis J Pollard JW. Macrophages: obligate partners for tumor cell migration, invasion, and metastasis. Cell (2006) 124:263–6. 10.1016/j.cell.2006.01.007 16439202

[67] Chen J Yao Y Gong C Yu F Su S Chen J. CCL18 from tumor-associated macrophages promotes breast cancer metastasis via PITPNM3. Cancer Cell (2011) 19:541–55. 10.1016/j.ccr.2011.02.006 21481794 PMC3107500

[68] Felbor U Dreier L Bryant RA Ploegh HL Olsen BR Mothes W. Secreted cathepsin L generates endostatin from collagen XVIII. EMBO J. (2000) 19:1187–94. 10.1093/emboj/19.6.1187 10716919 PMC305660

[69] Liu CY Xu JY Shi XY Huang W Ruan TY Xie P. M2-polarized tumor-associated macrophages promoted epithelial-mesenchymal transition in pancreatic cancer cells, partially through TLR4/IL-10 signaling pathway. Lab Invest. (2013) 93:844–54. 10.1038/labinvest.2013.69 23752129

[70] Su SC Liu Q Chen JQ Chen JN Chen F He CH. A positive feedback loop between mesenchymal-like cancer cells and macrophages is essential to breast cancer metastasis. Cancer Cell (2014) 25:605–20. 10.1016/j.ccr.2014.03.021 24823638

[71] Raggi C Mousa HS Correnti M Sica A Invernizzi P. Cancer stem cells and tumor-associated macrophages: a roadmap for multitargeting strategies. Oncogene (2016) 35:671–82. 10.1038/onc.2015.132 25961921

[72] Fan QM Jing YY Yu GF Kou XR Ye F Gao L. Tumor-associated macrophages promote cancer stem cell-like properties via transforming growth factor-beta1-induced epithelial-mesenchymal transition in hepatocellular carcinoma. Cancer Lett. (2014) 352:160–8. 10.1016/j.canlet.2014.05.008 24892648

[73] Kolaczkowska E Kubes P. Neutrophil recruitment and function in health and inflammation. Nat Rev Immunol. (2013) 13:159–75. 10.1038/nri3399 23435331

[74] Coffelt SB Wellenstein MD De Visser KE. Neutrophils in cancer: neutral no more. Nat Rev Cancer (2016) 16:431–46. 10.1038/nrc.2016.52 27282249

[75] Martins A Han J Kim SO. The multifaceted effects of granulocyte colony-stimulating factor in immunomodulation and potential roles in intestinal immune homeostasis. IUBMB Life (2010) 62:611–7. 10.1002/iub.361 20681025 PMC2916186

[76] Houghton AM Rzymkiewicz DM Ji H Gregory AD Egea EE Metz HE. Neutrophil elastase-mediated degradation of IRS-1 accelerates lung tumor growth. Nat Med. (2010) 16:219–23. 10.1038/nm.2084 20081861 PMC2821801

[77] Gong L Cumpian AM Caetano MS Ochoa CE De La Garza MM Lapid DJ. Promoting effect of neutrophils on lung tumorigenesis is mediated by CXCR2 and neutrophil elastase. Mol Cancer (2013) 12:154. 10.1186/1476-4598-12-154 24321240 PMC3923587

[78] Deryugina EI Zajac E Juncker-Jensen A Kupriyanova TA Welter L Quigley JP. Tissue-infiltrating neutrophils constitute the major *in vivo* source of angiogenesis-inducing MMP-9 in the tumor microenvironment. Neoplasia (2014) 16:771–88. 10.1016/j.neo.2014.08.013 25379015 PMC4212255

[79] Fridlender ZG Sun J Kim S Kapoor V Cheng G Ling L. Polarization of tumor-associated neutrophil phenotype by TGF-beta: “N1” versus “N2” TAN. Cancer Cell (2009) 16:183–94. 10.1016/j.ccr.2009.06.017 19732719 PMC2754404

[80] Moore RJ Owens DM Stamp G Arnott C Burke F East N. Mice deficient in tumor necrosis factor-alpha are resistant to skin carcinogenesis. Nat Med. (1999) 5:828–31. 10.1038/10552 10395330

[81] Zhou SL Dai Z Zhou ZJ Wang XY Yang GH Wang Z. Overexpression of CXCL5 mediates neutrophil infiltration and indicates poor prognosis for hepatocellular carcinoma. Hepatology (2012) 56:2242–54. 10.1002/hep.25907 22711685

[82] Coussens LM Tinkle CL Hanahan D Werb Z. MMP-9 supplied by bone marrow-derived cells contributes to skin carcinogenesis. Cell (2000) 103:481–90. 10.1016/S0092-8674(00)00139-2 11081634 PMC2843102

[83] Bekes EM Schweighofer B Kupriyanova TA Zajac E Ardi VC Quigley JP. Tumor-recruited neutrophils and neutrophil TIMP-free MMP-9 regulate coordinately the levels of tumor angiogenesis and efficiency of malignant cell intravasation. Am J Pathol. (2011) 179:1455–70. 10.1016/j.ajpath.2011.05.031 21741942 PMC3157227

[84] Grosse-Steffen T Giese T Giese N Longerich T Schirmacher P Hansch GM. Epithelial-to-mesenchymal transition in pancreatic ductal adenocarcinoma and pancreatic tumor cell lines: the role of neutrophils and neutrophil-derived elastase. Clin Dev Immunol. (2012) 2012:720768. 10.1155/2012/720768 23227088 PMC3514849

[85] Freisinger CM Huttenlocher A. Live imaging and gene expression analysis in zebrafish identifies a link between neutrophils and epithelial to mesenchymal transition. PLoS ONE (2014) 9:e112183. 10.1371/journal.pone.0112183 25372289 PMC4221567

[86] Jamieson T Clarke M Steele CW Samuel MS Neumann J Jung A. Inhibition of CXCR2 profoundly suppresses inflammation-driven and spontaneous tumorigenesis. J Clin Invest. (2012) 122:3127–44. 10.1172/JCI61067 22922255 PMC3428079

[87] He SN Lamers GEM Beenakker JWM Cui C Ghotra VPS Danen EHJ. Neutrophil-mediated experimental metastasis is enhanced by VEGFR inhibition in a zebrafish xenograft model. J Pathol. (2012) 227:431–45. 10.1002/path.4013 22374800 PMC3504093

[88] Glogauer JE Sun CX Bradley G Magalhaes MA. Neutrophils increase oral squamous cell carcinoma invasion through an invadopodia-dependent pathway. Cancer Immunol Res. (2015) 3:1218–26. 10.1158/2326-6066.CIR-15-0017 26112922

[89] Garcia-Mendoza MG Inman DR Ponik SM Jeffery JJ Sheerar DS Van Doorn RR. Neutrophils drive accelerated tumor progression in the collagen-dense mammary tumor microenvironment. Breast Cancer Res. (2016) 18:49. 10.1186/s13058-016-0703-7 27169366 PMC4864897

[90] Cools-Lartigue J Spicer J Najmeh S Ferri L. Neutrophil extracellular traps in cancer progression. Cell Mol Life Sci. (2014) 71:4179–94. 10.1007/s00018-014-1683-3 25070012 PMC7096049

[91] Erpenbeck L Schon MP. Neutrophil extracellular traps: protagonists of cancer progression? Oncogene (2017) 36:2483–90. 10.1038/onc.2016.406 27941879

[92] Cools-Lartigue J Spicer J Mcdonald B Gowing S Chow S Giannias B Neutrophil extracellular traps sequester circulating tumor cells and promote metastasis. J Clin Invest. (2013) 123:3446–58. 10.1172/JCI67484 PMC3726160 23863628

[93] Monti M Iommelli F De Rosa V Carriero MV Miceli R Camerlingo R. Integrin-dependent cell adhesion to neutrophil extracellular traps through engagement of fibronectin in neutrophil-like cells. PLoS ONE (2017) 12:e0171362. 10.1371/journal.pone.0171362 28166238 PMC5293257

[94] Sethi JK Vidal-Puig AJ. Thematic review series: adipocyte biology. Adipose tissue function and plasticity orchestrate nutritional adaptation. J Lipid Res. (2007) 48:1253–62. 10.1194/jlr.R700005-JLR200 17374880 PMC4303760

[95] Nieman KM Romero IL Van Houten B Lengyel E. Adipose tissue and adipocytes support tumorigenesis and metastasis. Biochim Biophys Acta (2013) 1831:1533–41. 10.1016/j.bbalip.2013.02.010 23500888 PMC3742583

[96] Duong MN Geneste A Fallone F Li X Dumontet C Muller C. The fat and the bad: Mature adipocytes, key actors in tumor progression and resistance. Oncotarget (2017) 8:57622–41. 10.18632/oncotarget.18038 28915700 PMC5593672

[97] Rodeheffer MS Birsoy K Friedman JM. Identification of white adipocyte progenitor cells *in vivo*. Cell (2008) 135:240–9. 10.1016/j.cell.2008.09.036 18835024

[98] Tang QQ Lane MD. Adipogenesis: from stem cell to adipocyte. Annu Rev Biochem. (2012) 81:715–36. 10.1146/annurev-biochem-052110-115718 22463691

[99] Tomiyama K Murase N Stolz DB Toyokawa H O'donnell DR Smith DM. Characterization of transplanted green fluorescent protein+ bone marrow cells into adipose tissue. Stem Cells (2008) 26:330–8. 10.1634/stemcells.2007-0567 17975222 PMC2268622

[100] Sera Y Larue AC Moussa O Mehrotra M Duncan JD Williams CR. Hematopoietic stem cell origin of adipocytes. Exp Hematol. (2009) 37:1108–20, 1120.e1–4. 10.1016/j.exphem.2009.06.008 19576951 PMC2740899

[101] Dirat B Bochet L Dabek M Daviaud D Dauvillier S Majed B. Cancer-associated adipocytes exhibit an activated phenotype and contribute to breast cancer invasion. Cancer Res. (2011) 71:2455–65. 10.1158/0008-5472.CAN-10-3323 21459803

[102] Bochet L Lehuede C Dauvillier S Wang YY Dirat B Laurent V. Adipocyte-derived fibroblasts promote tumor progression and contribute to the desmoplastic reaction in breast cancer. Cancer Res. (2013) 73:5657–68. 10.1158/0008-5472.CAN-13-0530 23903958

[103] Carter JC Church FC. Mature breast adipocytes promote breast cancer cell motility. Exp Mol Pathol. (2012) 92:312–7. 10.1016/j.yexmp.2012.03.005 22445926

[104] Nieman KM Kenny HA Penicka CV Ladanyi A Buell-Gutbrod R Zillhardt MR. Adipocytes promote ovarian cancer metastasis and provide energy for rapid tumor growth. Nat Med. (2011) 17:1498–503. 10.1038/nm.2492 22037646 PMC4157349

[105] Park J Morley TS Kim M Clegg DJ Scherer PE. Obesity and cancer–mechanisms underlying tumour progression and recurrence. Nat Rev Endocrinol. (2014) 10:455–65. 10.1038/nrendo.2014.94 24935119 PMC4374431

[106] Donohoe CL Lysaght J O'sullivan J Reynolds JV. Emerging concepts linking obesity with the hallmarks of cancer. Trends Endocrinol Metab. (2017) 28:46–62. 10.1016/j.tem.2016.08.004 27633129

[107] Incio J Liu H Suboj P Chin SM Chen IX Pinter M. Obesity-induced inflammation and desmoplasia promote pancreatic cancer progression and resistance to chemotherapy. Cancer Discov. (2016) 6:852–69. 10.1158/2159-8290.CD-15-1177 27246539 PMC4972679

[108] Seo BR Bhardwaj P Choi S Gonzalez J Andresen Eguiluz RC Wang K. Obesity-dependent changes in interstitial ECM mechanics promote breast tumorigenesis. Sci Transl Med. (2015) 7:301ra130. 10.1126/scitranslmed.3010467 26290412 PMC4837896

[109] Ribeiro RJ Monteiro CP Cunha VF Azevedo AS Oliveira MJ Monteiro R. Tumor cell-educated periprostatic adipose tissue acquires an aggressive cancer-promoting secretory profile. Cell Physiol Biochem. (2012) 29:233–40. 10.1159/000337604 22415092

[110] Iyengar P Espina V Williams TW Lin Y Berry D Jelicks LA. Adipocyte-derived collagen VI affects early mammary tumor progression *in vivo*, demonstrating a critical interaction in the tumor/stroma microenvironment. J Clin Invest. (2005) 115:1163–76. 10.1172/JCI23424 15841211 PMC1077173

[111] Park J Scherer PE. Adipocyte-derived endotrophin promotes malignant tumor progression. J Clin Invest. (2012) 122:4243–56. 10.1172/JCI63930 23041627 PMC3484450

[112] Campo-Verde-Arbocco F Lopez-Laur JD Romeo LR Giorlando N Bruna FA Contador DE. Human renal adipose tissue induces the invasion and progression of renal cell carcinoma. Oncotarget (2017) 8:94223–34. 10.18632/oncotarget.21666 29212223 PMC5706869

[113] De Visser KE Eichten A Coussens LM. Paradoxical roles of the immune system during cancer development. Nat Rev Cancer (2006) 6:24–37. 10.1038/nrc1782 16397525

[114] Sasada T Suekane S. Variation of tumor-infiltrating lymphocytes in human cancers: controversy on clinical significance. Immunotherapy (2011) 3:1235–51. 10.2217/imt.11.106 21995574

[115] Wakita D Sumida K Iwakura Y Nishikawa H Ohkuri T Chamoto K. Tumor-infiltrating IL-17-producing gammadelta T cells support the progression of tumor by promoting angiogenesis. Eur J Immunol. (2010) 40:1927–37. 10.1002/eji.200940157 20397212

[116] Coffelt SB Kersten K Doornebal CW Weiden J Vrijland K Hau CS. IL-17-producing gammadelta T cells and neutrophils conspire to promote breast cancer metastasis. Nature (2015) 522:345–8. 10.1038/nature14282 25822788 PMC4475637

[117] Denardo DG Barreto JB Andreu P Vasquez L Tawfik D Kolhatkar N. CD4(+) T cells regulate pulmonary metastasis of mammary carcinomas by enhancing protumor properties of macrophages. Cancer Cell (2009) 16:91–102. 10.1016/j.ccr.2009.06.018 19647220 PMC2778576

[118] Smyth MJ Teng MW Swann J Kyparissoudis K Godfrey DI Hayakawa Y. CD4+CD25+ T regulatory cells suppress NK cell-mediated immunotherapy of cancer. J Immunol. (2006) 176:1582–7. 10.4049/jimmunol.176.3.1582 16424187

[119] Facciabene A Peng X Hagemann IS Balint K Barchetti A Wang LP. Tumour hypoxia promotes tolerance and angiogenesis via CCL28 and T(reg) cells. Nature (2011) 475:226–30. 10.1038/nature10169 21753853

[120] De Visser KE Korets LV Coussens LM. De novo carcinogenesis promoted by chronic inflammation is B lymphocyte dependent. Cancer Cell (2005) 7:411–23. 10.1016/j.ccr.2005.04.014 15894262

[121] Ammirante M Luo JL Grivennikov S Nedospasov S Karin M. B-cell-derived lymphotoxin promotes castration-resistant prostate cancer. Nature (2010) 464:302–5. 10.1038/nature08782 20220849 PMC2866639

[122] Yang C Lee H Pal S Jove V Deng J Zhang W. B cells promote tumor progression via STAT3 regulated-angiogenesis. PLoS ONE (2013) 8:e64159. 10.1371/journal.pone.0064159 23734190 PMC3667024

[123] Edsparr K Basse PH Goldfarb RH Albertsson P. Matrix metalloproteinases in cytotoxic lymphocytes impact on tumour infiltration and immunomodulation. Cancer Microenviron. (2011) 4:351–60. 10.1007/s12307-010-0057-0 22161319 PMC3234320

[124] Johnatty RN Taub DD Reeder SP Turcovski-Corrales SM Cottam DW Stephenson TJ. Cytokine and chemokine regulation of proMMP-9 and TIMP-1 production by human peripheral blood lymphocytes. J Immunol. (1997) 158:2327–33. 9036981

[125] Segarra M Vilardell C Matsumoto K Esparza J Lozano E Serra-Pages C. Dual function of focal adhesion kinase in regulating integrin-induced MMP-2 and MMP-9 release by human T lymphoid cells. FASEB J. (2005) 19:1875–7. 10.1096/fj.04-3574fje 16260653

[126] Owen JL Iragavarapu-Charyulu V Gunja-Smith Z Herbert LM Grosso JF Lopez DM. Up-regulation of matrix metalloproteinase-9 in T lymphocytes of mammary tumor bearers: role of vascular endothelial growth factor. J Immunol. (2003) 171:4340–51. 10.4049/jimmunol.171.8.4340 14530359

[127] Mach F Schonbeck U Fabunmi RP Murphy C Atkinson E Bonnefoy JY. T lymphocytes induce endothelial cell matrix metalloproteinase expression by a CD40L-dependent mechanism: implications for tubule formation. Am J Pathol. (1999) 154:229–38. 10.1016/S0002-9440(10)65269-8 9916937 PMC1853443

[128] Aoudjit F Esteve PO Desrosiers M Potworowski EF St-Pierre Y. Gelatinase B (MMP-9) production and expression by stromal cells in the normal and adult thymus and experimental thymic lymphoma. Int J Cancer (1997) 71:71–8. 10.1002/(SICI)1097-0215(19970328)71:1<71::AID-IJC13>3.0.CO;2-C 9096668

[129] Casey T Bond J Tighe S Hunter T Lintault L Patel O. Molecular signatures suggest a major role for stromal cells in development of invasive breast cancer. Breast Cancer Res Treat (2009) 114:47–62. 10.1007/s10549-008-9982-8 18373191

[130] Naba A Clauser KR Hoersch S Liu H Carr SA Hynes RO. The matrisome: in silico definition and *in vivo* characterization by proteomics of normal and tumor extracellular matrices. Mol Cell Proteomics (2012) 11:M111.014647. 10.1074/mcp.M111.014647 22159717 PMC3322572

[131] Naba A Clauser KR Lamar JM Carr SA Hynes RO. Extracellular matrix signatures of human mammary carcinoma identify novel metastasis promoters. Elife (2014) 3:e01308. 10.7554/eLife.01308 24618895 PMC3944437

[132] Denko NC Fontana LA Hudson KM Sutphin PD Raychaudhuri S Altman R. Investigating hypoxic tumor physiology through gene expression patterns. Oncogene (2003) 22:5907–14. 10.1038/sj.onc.1206703 12947397

[133] Erler JT Bennewith KL Nicolau M Dornhofer N Kong C Le QT. Lysyl oxidase is essential for hypoxia-induced metastasis. Nature (2006) 440:1222–6. 10.1038/nature04695 16642001

[134] Levental KR Yu H Kass L Lakins JN Egeblad M Erler JT. Matrix crosslinking forces tumor progression by enhancing integrin signaling. Cell (2009) 139:891–906. 10.1016/j.cell.2009.10.027 19931152 PMC2788004

[135] Boyle ST Samuel MS. Mechano-reciprocity is maintained between physiological boundaries by tuning signal flux through the Rho-associated protein kinase. Small GTPases (2016) 7:139–46. 10.1080/21541248.2016.1173771 27168253 PMC5003540

[136] Boyle ST Kular J Nobis M Ruszkiewicz A Timpson P Samuel MS Acute compressive stress activates RHO/ROCK-mediated cellular processes. Small GTPases (2018) 17:1–17. 10.1080/21541248.2017.1413496 PMC7549670 29455593

[137] Gilkes DM Semenza GL Wirtz D. Hypoxia and the extracellular matrix: drivers of tumour metastasis. Nat Rev Cancer (2014) 14:430–9. 10.1038/nrc3726 24827502 PMC4283800

[138] Gilkes DM Chaturvedi P Bajpai S Wong CC Wei H Pitcairn S. Collagen prolyl hydroxylases are essential for breast cancer metastasis. Cancer Res. (2013) 73:3285–96. 10.1158/0008-5472.CAN-12-3963 23539444 PMC3674184

[139] Gilkes DM Bajpai S Wong CC Chaturvedi P Hubbi ME Wirtz D. Procollagen lysyl hydroxylase 2 is essential for hypoxia-induced breast cancer metastasis. Mol Cancer Res. (2013) 11:456–66. 10.1158/1541-7786.MCR-12-0629 23378577 PMC3656974

[140] Krishnamachary B Berg-Dixon S Kelly B Agani F Feldser D Ferreira G. Regulation of colon carcinoma cell invasion by hypoxia-inducible factor 1. Cancer Res. (2003) 63:1138–43. 12615733

[141] Munoz-Najar UM Neurath KM Vumbaca F Claffey KP. Hypoxia stimulates breast carcinoma cell invasion through MT1-MMP and MMP-2 activation. Oncogene (2006) 25:2379–92. 10.1038/sj.onc.1209273 16369494

[142] Wolf K Wu YI Liu Y Geiger J Tam E Overall C. Multi-step pericellular proteolysis controls the transition from individual to collective cancer cell invasion. Nat Cell Biol. (2007) 9:893–904. 10.1038/ncb1616 17618273

[143] Ota I Li XY Hu Y Weiss SJ. Induction of a MT1-MMP and MT2-MMP-dependent basement membrane transmigration program in cancer cells by Snail1. Proc Natl Acad Sci USA. (2009) 106:20318–23. 10.1073/pnas.0910962106 19915148 PMC2787166

[144] Radisky ES Radisky DC. Matrix metalloproteinase-induced epithelial-mesenchymal transition in breast cancer. J Mammary Gland Biol Neoplasia (2010) 15:201–12. 10.1007/s10911-010-9177-x 20440544 PMC2886087

[145] Rath N Morton JP Julian L Helbig L Kadir S Mcghee EJ. ROCK signaling promotes collagen remodeling to facilitate invasive pancreatic ductal adenocarcinoma tumor cell growth. EMBO Mol Med. (2017) 9:198–218. 10.15252/emmm.201606743 28031255 PMC5286371

[146] Clancy JW Sedgwick A Rosse C Muralidharan-Chari V Raposo G Method M. Regulated delivery of molecular cargo to invasive tumour-derived microvesicles. Nat Commun. (2015) 6:6919. 10.1038/ncomms7919 25897521 PMC4497525

[147] Bordeleau F Chan B Antonyak MA Lampi MC Cerione RA Reinhart-King CA. Microvesicles released from tumor cells disrupt epithelial cell morphology and contractility. J Biomech. (2016) 49:1272–9. 10.1016/j.jbiomech.2015.10.003 26477404 PMC4826648

[148] Tkach M Thery C. Communication by extracellular vesicles: where we are and where we need to go. Cell (2016) 164:1226–32. 10.1016/j.cell.2016.01.043 26967288

[149] Mcniven MA. Breaking away: matrix remodeling from the leading edge. Trends Cell Biol. (2013) 23:16–21. 10.1016/j.tcb.2012.08.009 22999190 PMC3905740

[150] Albiges-Rizo C Destaing O Fourcade B Planus E Block MR. Actin machinery and mechanosensitivity in invadopodia, podosomes and focal adhesions. J Cell Sci. (2009) 122:3037–49. 10.1242/jcs.052704 19692590 PMC2767377

[151] Geiger B Yamada KM. Molecular architecture and function of matrix adhesions. Cold Spring Harb Perspect Biol. (2011) 3:a005033. 10.1101/cshperspect.a005033 21441590 PMC3101841

[152] Wang Y Mcniven MA. Invasive matrix degradation at focal adhesions occurs via protease recruitment by a FAK-p130Cas complex. J Cell Biol. (2012) 196:375–85. 10.1083/jcb.201105153 22291036 PMC3275373

[153] Jacob A Prekeris R. The regulation of MMP targeting to invadopodia during cancer metastasis. Front Cell Dev Biol. (2015) 3:4. 10.3389/fcell.2015.00004 25699257 PMC4313772

[154] Leong HS Robertson AE Stoletov K Leith SJ Chin CA Chien AE. Invadopodia are required for cancer cell extravasation and are a therapeutic target for metastasis. Cell Rep. (2014) 8:1558–70. 10.1016/j.celrep.2014.07.050 25176655

[155] Goetz JG Minguet S Navarro-Lerida I Lazcano JJ Samaniego R Calvo E. Biomechanical remodeling of the microenvironment by stromal caveolin-1 favors tumor invasion and metastasis. Cell (2011) 146:148–63. 10.1016/j.cell.2011.05.040 21729786 PMC3244213

[156] Scott RW Hooper S Crighton D Li A Konig I Munro J. LIM kinases are required for invasive path generation by tumor and tumor-associated stromal cells. J Cell Biol. (2010) 191:169–85. 10.1083/jcb.201002041 20876278 PMC2953444

[157] Boire A Covic L Agarwal A Jacques S Sherifl S Kuliopulos A. PAR1 is a matrix metalloprotease-1 receptor that promotes invasion and tumorigenesis of breast cancer cells. Cell (2005) 120:303–13. 10.1016/j.cell.2004.12.018 15707890

[158] Gocheva V Wang HW Gadea BB Shree T Hunter KE Garfall AL. IL-4 induces cathepsin protease activity in tumor-associated macrophages to promote cancer growth and invasion. Genes Dev. (2010) 24:241–55. 10.1101/gad.1874010 20080943 PMC2811826

[159] Faurobert E Bouin AP Albiges-Rizo C. Microenvironment, tumor cell plasticity, and cancer. Curr Opin Oncol. (2015) 27:64–70. 10.1097/CCO.0000000000000154 25415136

[160] Massague J Xi Q. TGF-beta control of stem cell differentiation genes. FEBS Lett. (2012) 586:1953–8. 10.1016/j.febslet.2012.03.023 22710171 PMC3466472

[161] Heldin CH Miyazono K Ten Dijke P. TGF-beta signalling from cell membrane to nucleus through SMAD proteins. Nature (1997) 390:465–71. 10.1038/37284 9393997

[162] Shi Y Massague J. Mechanisms of TGF-beta signaling from cell membrane to the nucleus. Cell (2003) 113:685–700. 10.1016/S0092-8674(03)00432-X 12809600

[163] Sun PD Davies DR. The cystine-knot growth-factor superfamily. Annu Rev Biophys Biomol Struct. (1995) 24:269–91. 10.1146/annurev.bb.24.060195.001413 7663117

[164] Assoian RK Fleurdelys BE Stevenson HC Miller PJ Madtes DK Raines EW. Expression and secretion of type beta transforming growth factor by activated human macrophages. Proc Natl Acad Sci USA. (1987) 84:6020–4. 10.1073/pnas.84.17.6020 2888109 PMC298999

[165] Schultz-Cherry S Murphy-Ullrich JE. Thrombospondin causes activation of latent transforming growth factor-beta secreted by endothelial cells by a novel mechanism. J Cell Biol. (1993) 122:923–32. 10.1083/jcb.122.4.923 8349738 PMC2119591

[166] Kelley J Fabisiak JP Hawes K Absher M. Cytokine signaling in lung: transforming growth factor-beta secretion by lung fibroblasts. Am J Physiol. (1991) 260:L123–128. 1996655 10.1152/ajplung.1991.260.2.L123

[167] Massague J. TGFbeta signalling in context. Nat Rev Mol Cell Biol. (2012) 13:616–30. 10.1038/nrm3434 22992590 PMC4027049

[168] Cui W Fowlis DJ Bryson S Duffie E Ireland H Balmain A. TGFbeta1 inhibits the formation of benign skin tumors, but enhances progression to invasive spindle carcinomas in transgenic mice. Cell (1996) 86:531–42. 10.1016/S0092-8674(00)80127-0 8752208

[169] Oft M Heider KH Beug H. TGFbeta signaling is necessary for carcinoma cell invasiveness and metastasis. Curr Biol. (1998) 8:1243–52. 10.1016/S0960-9822(07)00533-7 9822576

[170] Muraoka RS Dumont N Ritter CA Dugger TC Brantley DM Chen J. Blockade of TGF-beta inhibits mammary tumor cell viability, migration, and metastases. J Clin Invest. (2002) 109:1551–9. 10.1172/JCI0215234 12070302 PMC151012

[171] Ischenko I Liu J Petrenko O Hayman MJ. Transforming growth factor-beta signaling network regulates plasticity and lineage commitment of lung cancer cells. Cell Death Differ. (2014) 21:1218–28. 10.1038/cdd.2014.38 24682004 PMC4085528

[172] Taylor MA Amin JD Kirschmann DA Schiemann WP. Lysyl oxidase contributes to mechanotransduction-mediated regulation of transforming growth factor-beta signaling in breast cancer cells. Neoplasia (2011) 13:406–18. 10.1593/neo.101086 21532881 PMC3084617

[173] Cox TR Bird D Baker AM Barker HE Ho MW Lang G. LOX-mediated collagen crosslinking is responsible for fibrosis-enhanced metastasis. Cancer Res. (2013) 73:1721–32. 10.1158/0008-5472.CAN-12-2233 23345161 PMC3672851

[174] Taipale J Miyazono K Heldin CH Keski-Oja J. Latent transforming growth factor-beta 1 associates to fibroblast extracellular matrix via latent TGF-beta binding protein. J Cell Biol. (1994) 124:171–81. 10.1083/jcb.124.1.171 8294500 PMC2119892

[175] Babic AM Chen CC Lau LF. Fisp12/mouse connective tissue growth factor mediates endothelial cell adhesion and migration through integrin alphavbeta3, promotes endothelial cell survival, and induces angiogenesis *in vivo*. Mol Cell Biol. (1999) 19:2958–66. 10.1128/MCB.19.4.2958 10082563 PMC84090

[176] Abreu JG Ketpura NI Reversade B De Robertis EM. Connective-tissue growth factor (CTGF) modulates cell signalling by BMP and TGF-beta. Nat Cell Biol. (2002) 4:599–604. 10.1038/ncb826 12134160 PMC2387275

[177] Chang CC Hsu WH Wang CC Chou CH Kuo MY Lin BR. Connective tissue growth factor activates pluripotency genes and mesenchymal-epithelial transition in head and neck cancer cells. Cancer Res. (2013) 73:4147–57. 10.1158/0008-5472.CAN-12-4085 23687336

[178] Zeng H Yang Z Xu N Liu B Fu Z Lian C. Connective tissue growth factor promotes temozolomide resistance in glioblastoma through TGF-beta1-dependent activation of Smad/ERK signaling. Cell Death Dis. (2017) 8:e2885. 10.1038/cddis.2017.248 28617438 PMC5520906

[179] Finger EC Cheng CF Williams TR Rankin EB Bedogni B Tachiki L. CTGF is a therapeutic target for metastatic melanoma. Oncogene (2014) 33:1093–100. 10.1038/onc.2013.47 23435419 PMC3965577

[180] Tamai K Semenov M Kato Y Spokony R Liu C Katsuyama Y. LDL-receptor-related proteins in Wnt signal transduction. Nature (2000) 407:530–5. 10.1038/35035117 11029007

[181] Bhanot P Brink M Samos CH Hsieh JC Wang Y Macke JP. A new member of the frizzled family from Drosophila functions as a Wingless receptor. Nature (1996) 382:225–30. 10.1038/382225a0 8717036

[182] Itoh K Krupnik VE Sokol SY. Axis determination in Xenopus involves biochemical interactions of axin, glycogen synthase kinase 3 and beta-catenin. Curr Biol. (1998) 8:591–4. 10.1016/S0960-9822(98)70229-5 9601644

[183] Kishida S Yamamoto H Hino S Ikeda S Kishida M Kikuchi A. DIX domains of Dvl and axin are necessary for protein interactions and their ability to regulate beta-catenin stability. Mol Cell Biol. (1999) 19:4414–22. 10.1128/MCB.19.6.4414 10330181 PMC104400

[184] Smalley MJ Sara E Paterson H Naylor S Cook D Jayatilake H. Interaction of axin and Dvl-2 proteins regulates Dvl-2-stimulated TCF-dependent transcription. Embo J. (1999) 18:2823–35. 10.1093/emboj/18.10.2823 10329628 PMC1171363

[185] Rubinfeld B Albert I Porfiri E Fiol C Munemitsu S Polakis P. Binding of GSK3beta to the APC-beta-catenin complex and regulation of complex assembly. Science (1996) 272:1023–6. 10.1126/science.272.5264.1023 8638126

[186] Yost C Torres M Miller JR Huang E Kimelman D Moon RT. The axis-inducing activity, stability, and subcellular distribution of beta-catenin is regulated in Xenopus embryos by glycogen synthase kinase 3. Genes Dev. (1996) 10:1443–54. 10.1101/gad.10.12.1443 8666229

[187] Hart MJ De Los Santos R Albert IN Rubinfeld B Polakis P. Downregulation of beta-catenin by human Axin and its association with the APC tumor suppressor, beta-catenin and GSK3 beta. Curr Biol. (1998) 8:573–81. 10.1016/S0960-9822(98)70226-X 9601641

[188] Yamamoto H Kishida S Kishida M Ikeda S Takada S Kikuchi A. Phosphorylation of axin, a Wnt signal negative regulator, by glycogen synthase kinase-3beta regulates its stability. J Biol Chem. (1999) 274:10681–4. 10.1074/jbc.274.16.10681 10196136

[189] Aberle H Bauer A Stappert J Kispert A Kemler R. beta-catenin is a target for the ubiquitin-proteasome pathway. Embo J. (1997) 16:3797–804. 10.1093/emboj/16.13.3797 9233789 PMC1170003

[190] Marikawa Y Elinson RP. beta-TrCP is a negative regulator of Wnt/beta-catenin signaling pathway and dorsal axis formation in Xenopus embryos. Mech Dev. (1998) 77:75–80. 10.1016/S0925-4773(98)00134-8 9784611

[191] Behrens J Von Kries JP Kuhl M Bruhn L Wedlich D Grosschedl R. Functional interaction of beta-catenin with the transcription factor LEF-1. Nature (1996) 382:638–42. 10.1038/382638a0 8757136

[192] Roose J Molenaar M Peterson J Hurenkamp J Brantjes H Moerer P. The Xenopus Wnt effector XTcf-3 interacts with Groucho-related transcriptional repressors. Nature (1998) 395:608–12. 10.1038/26989 9783587

[193] Waltzer L Bienz M. Drosophila CBP represses the transcription factor TCF to antagonize Wingless signalling. Nature (1998) 395:521–5. 10.1038/26785 9774110

[194] Brannon M Brown JD Bates R Kimelman D Moon RT. XCtBP is a XTcf-3 co-repressor with roles throughout Xenopus development. Development (1999) 126:3159–70. 10375506 10.1242/dev.126.14.3159

[195] Brantjes H Roose J Van De Wetering M Clevers H. All Tcf HMG box transcription factors interact with Groucho-related co-repressors. Nucleic Acids Res. (2001) 29:1410–9. 10.1093/nar/29.7.1410 11266540 PMC31284

[196] Yook JI Li XY Ota I Hu C Kim HS Kim NH. A Wnt-Axin2-GSK3beta cascade regulates Snail1 activity in breast cancer cells. Nat Cell Biol. (2006) 8:1398–406. 10.1038/ncb1508 17072303

[197] Conacci-Sorrell M Simcha I Ben-Yedidia T Blechman J Savagner P Ben-Ze'ev A. Autoregulation of E-cadherin expression by cadherin-cadherin interactions: the roles of beta-catenin signaling, Slug, and MAPK. J Cell Biol. (2003) 163:847–57. 10.1083/jcb.200308162 14623871 PMC2173691

[198] Howe LR Watanabe O Leonard J Brown AM. Twist is up-regulated in response to Wnt1 and inhibits mouse mammary cell differentiation. Cancer Res. (2003) 63:1906–13. 12702582

[199] Dimeo TA Anderson K Phadke P Fan C Perou CM Naber S. A novel lung metastasis signature links Wnt signaling with cancer cell self-renewal and epithelial-mesenchymal transition in basal-like breast cancer. Cancer Res. (2009) 69:5364–73. 10.1158/0008-5472.CAN-08-4135 19549913 PMC2782448

[200] Xu W Wang Z Zhang W Qian K Li H Kong D. Mutated K-ras activates CDK8 to stimulate the epithelial-to-mesenchymal transition in pancreatic cancer in part via the Wnt/beta-catenin signaling pathway. Cancer Lett. (2015) 356:613–27. 10.1016/j.canlet.2014.10.008 25305448

[201] Gujral TS Chan M Peshkin L Sorger PK Kirschner MW Macbeath G. A noncanonical Frizzled2 pathway regulates epithelial-mesenchymal transition and metastasis. Cell (2014) 159:844–56. 10.1016/j.cell.2014.10.032 25417160 PMC4243058

[202] Pinto D Gregorieff A Begthel H Clevers H. Canonical Wnt signals are essential for homeostasis of the intestinal epithelium. Genes Dev. (2003) 17:1709–13. 10.1101/gad.267103 12865297 PMC196179

[203] Bastide P Darido C Pannequin J Kist R Robine S Marty-Double C. Sox9 regulates cell proliferation and is required for Paneth cell differentiation in the intestinal epithelium. J Cell Biol. (2007) 178:635–48. 10.1083/jcb.200704152 17698607 PMC2064470

[204] Goessling W North TE Loewer S Lord AM Lee S Stoick-Cooper CL. Genetic interaction of PGE2 and Wnt signaling regulates developmental specification of stem cells and regeneration. Cell (2009) 136:1136–47. 10.1016/j.cell.2009.01.015 19303855 PMC2692708

[205] Du J Zu Y Li J Du S Xu Y Zhang L. Extracellular matrix stiffness dictates Wnt expression through integrin pathway. Sci Rep. (2016) 6:20395. 10.1038/srep20395 26854061 PMC4745056

[206] Etienne-Manneville S Hall A. Rho GTPases in cell biology. Nature (2002) 420:629–35. 10.1038/nature01148 12478284

[207] Samuel MS Olson MF ROCK. In: Choi S, editor. Encyclopedia of Signaling Molecules. Cham: Springer International Publishing (2018) 4746–51. 10.1007/978-3-319-67199-4_328

[208] Leung T Manser E Tan L Lim L. A novel serine/threonine kinase binding the Ras-related RhoA GTPase which translocates the kinase to peripheral membranes. J Biol Chem. (1995) 270:29051–4. 10.1074/jbc.270.49.29051 7493923

[209] Ishizaki T Maekawa M Fujisawa K Okawa K Iwamatsu A Fujita A. The small GTP-binding protein Rho binds to and activates a 160 kDa Ser/Thr protein kinase homologous to myotonic dystrophy kinase. Embo J. (1996) 15:1885–93. 10.1002/j.1460-2075.1996.tb00539.x 8617235 PMC450107

[210] Matsui T Amano M Yamamoto T Chihara K Nakafuku M Ito M. Rho-associated kinase, a novel serine/threonine kinase, as a putative target for small GTP binding protein Rho. Embo J. (1996) 15:2208–16. 10.1002/j.1460-2075.1996.tb00574.x 8641286 PMC450144

[211] Scott RW Olson MF. LIM kinases: function, regulation and association with human disease. J Mol Med (Berl). (2007) 85:555–68. 10.1007/s00109-007-0165-6 17294230

[212] Croft DR Crighton D Samuel MS Lourenco FC Munro J Wood J. p53-mediated transcriptional regulation and activation of the actin cytoskeleton regulatory RhoC to LIMK2 signaling pathway promotes cell survival. Cell Res. (2011) 21:666–82. 10.1038/cr.2010.154 21079653 PMC3145139

[213] Olson MF Sahai E. The actin cytoskeleton in cancer cell motility. Clin Exp Meta. (2009) 26:273–87. 10.1007/s10585-008-9174-2 18498004

[214] Narumiya S Tanji M Ishizaki T. Rho signaling, ROCK and mDia1, in transformation, metastasis and invasion. Cancer Metastasis Rev. (2009) 28:65–76. 10.1007/s10555-008-9170-7 19160018

[215] Woodcock JM Coolen C Goodwin KL Baek DJ Bittman R Samuel MS. Destabilisation of dimeric 14–3-3 proteins as a novel approach to anti-cancer therapeutics. Oncotarget (2015) 6:14522–36. 10.18632/oncotarget.3995 25971334 PMC4546484

[216] Harvey KF Pfleger CM Hariharan IK. The Drosophila Mst ortholog, hippo, restricts growth and cell proliferation and promotes apoptosis. Cell (2003) 114:457–67. 10.1016/S0092-8674(03)00557-9 12941274

[217] Vassilev A Kaneko KJ Shu H Zhao Y Depamphilis ML. TEAD/TEF transcription factors utilize the activation domain of YAP65, a Src/Yes-associated protein localized in the cytoplasm. Genes Dev. (2001) 15:1229–41. 10.1101/gad.888601 11358867 PMC313800

[218] Dupont S Morsut L Aragona M Enzo E Giulitti S Cordenonsi M. Role of YAP/TAZ in mechanotransduction. Nature (2011) 474:179–83. 10.1038/nature10137 21654799

[219] Panciera T Azzolin L Cordenonsi M Piccolo S. Mechanobiology of YAP and TAZ in physiology and disease. Nat Rev Mol Cell Biol. (2017) 18:758–70. 10.1038/nrm.2017.87 28951564 PMC6192510

[220] Diepenbruck M Waldmeier L Ivanek R Berninger P Arnold P Van Nimwegen E. Tead2 expression levels control the subcellular distribution of Yap and Taz, zyxin expression and epithelial-mesenchymal transition. J Cell Sci. (2014) 127:1523–36. 10.1242/jcs.139865 24554433

[221] Kim W Khan SK Gvozdenovic-Jeremic J Kim Y Dahlman J Kim H. Hippo signaling interactions with Wnt/beta-catenin and Notch signaling repress liver tumorigenesis. J Clin Invest. (2017) 127:137–52. 10.1172/JCI88486 27869648 PMC5199712

[222] Campos LS Decker L Taylor V Skarnes W. Notch, epidermal growth factor receptor, and beta1-integrin pathways are coordinated in neural stem cells. J Biol Chem. (2006) 281:5300–9. 10.1074/jbc.M511886200 16332675

[223] Quail DF Taylor MJ Postovit LM. Microenvironmental regulation of cancer stem cell phenotypes. Curr Stem Cell Res Ther. (2012) 7:197–216. 10.2174/157488812799859838 22329582

[224] Shirakihara T Horiguchi K Miyazawa K Ehata S Shibata T Morita I. TGF-beta regulates isoform switching of FGF receptors and epithelial-mesenchymal transition. EMBO J. (2011) 30:783–95. 10.1038/emboj.2010.351 21224849 PMC3041949

[225] Spina A De Pasquale V Cerulo G Cocchiaro P Della Morte R Avallone L. HGF/c-MET axis in tumor microenvironment and metastasis formation. Biomedicines (2015) 3:71–88. 10.3390/biomedicines3010071 28536400 PMC5344235

